# The Small GTPase RHOA Links SLP65 Activation to PTEN Function in Pre B Cells and Is Essential for the Generation and Survival of Normal and Malignant B Cells

**DOI:** 10.3389/fimmu.2022.842340

**Published:** 2022-03-15

**Authors:** Anila Vadakumchery, Hemin Faraidun, Omar El Ayoubi, Issame Outaleb, Vera Schmid, Hend Abdelrasoul, Timm Amendt, Ahmad Khadour, Corinna Setz, Katharina Göhring, Karoline Lodd, Christoffer Hitzing, Alabbas Alkhatib, Mayas Bilal, Julian Benckendorff, Abdul Kader Al Shugri, Cord Herbert Brakebusch, Niklas Engels, Moumita Datta, Elias Hobeika, Ameera Alsadeq, Hassan Jumaa

**Affiliations:** ^1^ Institute of Immunology, Ulm University Medical Center, Ulm, Germany; ^2^ Department of Molecular Immunology, Faculty of Biology, Albert Ludwigs University of Freiburg, Freiburg, Germany; ^3^ Institute of Cellular & Molecular Immunology, University Medical Center Göttingen, Göttingen, Germany; ^4^ Institute of Pathology, Ulm University Medical Center, Ulm, Germany; ^5^ Biotech Research and Innovation Center (BRIC), University of Copenhagen, Copenhagen, Denmark

**Keywords:** RHOA, PTEN, SLP65, PI3K signaling, BCR-ABL, CLL

## Abstract

The generation, differentiation, survival and activation of B cells are coordinated by signals emerging from the B cell antigen receptor (BCR) or its precursor, the pre-BCR. The adaptor protein SLP65 (also known as BLNK) is an important signaling factor that controls pre-B cell differentiation by down-regulation of PI3K signaling. Here, we investigated the mechanism by which SLP65 interferes with PI3K signaling. We found that SLP65 induces the activity of the small GTPase RHOA, which activates PTEN, a negative regulator of PI3K signaling, by enabling its translocation to the plasma membrane. The essential role of RHOA is confirmed by the complete block in early B cell development in conditional *RhoA*-deficient mice. The *RhoA*-deficient progenitor B cells showed defects in activation of immunoglobulin gene rearrangement and fail to survive both *in vitro* and *in vivo*. Reconstituting the *RhoA*-deficient cells with *RhoA* or *Foxo1*, a transcription factor repressed by PI3K signaling and activated by PTEN, completely restores the survival defect. However, the defect in differentiation can only be restored by *RhoA* suggesting a unique role for RHOA in B cell generation and selection. In full agreement, conditional RhoA-deficient mice develop increased amounts of autoreactive antibodies with age. RHOA function is also required at later stage, as inactivation of *RhoA* in peripheral B cells or in a transformed mature B cell line resulted in cell loss. Together, these data show that RHOA is the key signaling factor for B cell development and function by providing a crucial SLP65-activated link between BCR signaling and activation of PTEN. Moreover, the identified essential role of RHOA for the survival of transformed B cells offers the opportunity for targeting B cell malignancies by blocking RHOA function.

## Introduction

B lymphocyte development is a tightly synchronized process that is characterized by the sequential rearrangement of immunoglobulin gene loci, which leads to the expression of BCR. Successful V(D)J rearrangement at immunoglobulin (Ig) heavy chain (HC) locus in the pro B cell stage leads to the assembly of the pre-BCR complex on the surface of developing B cells (1–3). Pre-B cells with functional pre BCR undergo several stages of clonal proliferation and later rearrangement of immunoglobulin light chain (LC) locus, which allows them to differentiate to BCR expressing immature B cells (4, 5). Previously, our group and others have shown that the PI3K-AKT-FOXO pathway is important for proliferation as well as differentiation of the developing B cells. The pre-BCR dependent activation of Phosphoinositide 3 kinase (PI3K) leads to the recruitment of pleckstrin homology (PH) domain-containing signaling molecules such as AKT (also known as PKB) to the plasma membrane (6–8). AKT mediated phosphorylation of the forkhead box class O (FOXO) transcription factors results in their nuclear export and degradation (9–12). Several studies including ours have demonstrated that FOXO1, a B cell-specific FOXO family member, regulates *Ig* gene recombination through the induction of Recombination activating gene 1 and 2 (13–15). Moreover, FOXO1 activity is also dependent on Phosphatase and Tensin homolog (PTEN), a lipid phosphatase that antagonizes PI3K signaling (16–19). Another key component that is activated downstream of pre-BCR/BCR signaling is Src homology containing leucocyte protein of 65 kDa (SLP65) (20). SLP65 is a B cell-specific adaptor protein that down-regulates PI3K activity, thereby stabilizing FOXO transcription factors and inducing differentiation of pre-B cells (13). It is elusive how SLP65 interferes with the PI3K-AKT signaling pathway to activate FOXO proteins. We already showed the importance of N-terminal leucine zipper of SLP-65 for its recruitment and activation mechanism (20, 21), but an interaction partner that helps SLP-65 to translocate into the vicinity of pre-BCR at cell membrane was missing.

B cell development and immune response require proper cytoskeletal reorganization, migration, proliferation, and survival of B cells. One of the key players in this process is Rho GTPase, which transduces signals from multiple receptors (22, 23). Ras homolog gene family member A (RHOA) is an intracellular signal-transducing protein of the Rho family of small GTPases (24, 25). Guanine nucleotide exchange factors (GEFs) activate Rho GTPases by promoting the exchange of GDP for GTP (24, 26, 27). Upon activation by GEFs, RHOA binds to downstream effector molecules such as RhoA Associated Coiled-Coil Containing Protein Kinases1/2 (ROCK1/2) and modulates many cellular events (23).

RHOA plays a critical role both in early and late stages of B cell development. It has been shown that deletion of RHOA in hematopoietic stem cells leads to an increase in common lymphoid progenitor (CLP) together with a reduction of Pro/Pre/Immature B cell compartments in bone marrow (28). B cell specific deletion of RHOA using CD19-Cre impaired mature B cell development in spleen and also B cell activation factor (BAFF) mediated survival of B cells (28). Other three closely related Rho GTPases such as RAC1, RAC2, and CDC42 are also shown to be important for B cell differentiation and proper immune response despite having different effectors and molecular mechanisms (29, 30). Mature B cells require signals generated from BCR for their survival, proliferation and differentiation. Several studies have shown that RHOA and other Rho GTPases are activated downstream of PI3K upon BCR engagement and transduce signals that are required for the proliferation and survival of B cells (29–31). They are also demonstrated to be important for effective PI3K activation (32). Conversely RHOA is also shown to counteract PI3K signaling by regulating the localization and activity of PTEN *via* ROCK (33, 34). RHOA mediated Rock pathways are emerging as critical regulators in proper germinal center (GC) response and antigen-specific immunoglobulin production in B cells. Recently ROCK2, one of Rho effector kinase is shown to activate FOXO1, a key player in GC reaction and thereby regulating the formation and maintenance of GC (35). Apart from the role in normal B cell development, RhoA and its downstream targets ROCKs are found deregulated in many hematopoietic and non-hematopoietic malignancies (36–39). Hyperactivation of Rho kinases is observed in myeloid leukaemias and myeloproliferative diseases harbouring activating mutations (KIT, FLT3 and BCR-ABL) and is shown to be dependent on RHOA (40). On the other hand, inactivating mutations or deletions of RHOA are observed in B and T cell lymphomas (41). Analysis of RHOA involvement in leukemia and lymphomas from published literature and open source databases shows alteration of RHOA in 1.7% cases mainly by inactivating mutations or deletions while RHOB, RHOC and effector molecules ROCK1 and ROCK2 are not altered (42, 43). This suggests a key role of RHOA in the hematopoietic malignancies and therefore warrants further investigation.

In this study, we investigated the mechanism how RHOA is connected to B cell signaling thereby affecting B cell development and activation. Our data demonstrate that RHOA is a novel interaction partner of SLP65, which connects SLP65 activity to PI3K signaling pathway regulation. Moreover, we found that SLP65-mediated RHOA activation is required for the proper development and function of mature B cells and the generation of antibody responses. This study also provides evidence for a non-redundant role of RHOA in the development of B cell leukaemia in an *in vivo* mouse model.

## Results

### GEF-H1 Is A Novel Interaction Partner of SLP65

**Figure 1:**
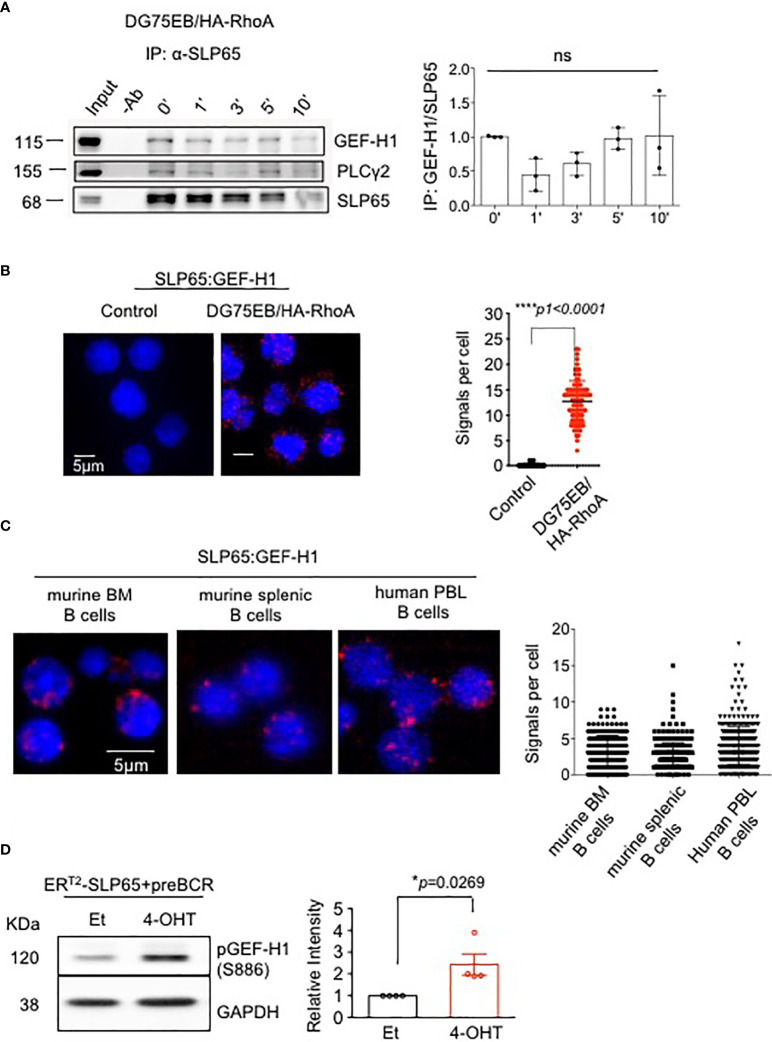
SLP65 interacts specifically with GEF-H1 and triggers its activation. **(A)** Left panel- Immunoprecipitation followed by western blot analysis for the interaction of endogenous SLP65 with GEF-H1 in DG75EB/HA-RhoA cells upon BCR stimulation. Cells were stimulated with anti-IgM antibody for the indicated time period and IP was carried out with anti-SLP65 antibody. PLCγ2, a known interactor of SLP65 is used as a positive control. Result representative of 3 independent experiments. Right Panel- Quantification of immunoprecipitated GEF-H1 with respect to SLP65. Statistical analysis- One-way ANOVA. **(B)** Left- Proximity ligation assay (PLA) to detect the association of SLP65 and GEF-H1 in unstimulated DG75EB/HA-RhoA cell line. Only secondary antibodies are used in the controls. Close proximity is shown as red dots. Right- quantification of number of signals per cell, error bars represent mean ± SD. Unpaired t-test, two-tailed. **(C)** PLA to detect the association of SLP65 and GEF-H1 in murine B cells isolated from bone marrow (BM) and spleen (SP) and human peripheral blood (PBL) B cells. B cells are isolated by CD19 magnetic sorting. Close proximity in a range of 10-40 nm is shown as red dots. Right- quantification of number of signals per cell, error bars represent mean ± SD. **(D)** Left- western blot analysis for the detection of phosphorylated GEF-H1 upon SLP65 activation in bone marrow derived early pre-B cells from *Rag2^-/-^*, *λ5^-/-^*, *Slp65^-/-^* triple knock out (TKO) mice. The cells were reconstituted with µ-heavy chain, λ5 and inducible SLP65-ER^T2^ construct and then induced with 2µM 4-hydroxy tamoxifen (4-OHT) or ethanol (Et) for 30 min to activate SLP65. GAPDH is used as loading control. Right- quantification of pGEF-H1 band intensity with respect to GAPDH. Result representative of 4 independent experiments. Statistical analysis- unpaired t test, two tailed. *p<0.05, ****p<0.0001, ns, not significant.

To explore the molecular mechanism of SLP65-mediated B cell regulation, we first used a split ubiquitin yeast-two-hybrid assay (Y2H) (44). The N-terminus of SLP65 (N-SLP65) was used to identify novel SLP65 interaction partners that might link SLP65 activity to other signaling molecules such as PI3K pathway proteins. The assay showed 10 potential candidates that may interact with N-SLP65 (**Table 1**). Among these potential partners is the RHOA guanine nucleotide exchange factor GEF-2 (ARHGEF2, also known as GEF-H1). Since small GTPases were previously shown to play an essential role in B cell development (28), we hypothesized that GEF-H1 could also be important in SLP65-mediated signaling. To validate our observation in the Y2H assay, we performed immunoprecipitation (IP) assay to see if the interaction takes place in endogenous protein level. To this end, we used human Burkitt lymphoma derived cell line DG75EB that overexpress HA tagged RHOA (DG75EB/HA-RhoA, see methods for details). The cells were stimulated with anti-human IgM F(ab)’2 antibody for different time points to activate SLP65 and IP was carried out using anti-SLP65 antibody. The interaction of SLP65 with GEF-H1 was detectable at similar extent at all time points including unstimulated condition (**Figure 1A**). As a positive control of IP, we also detected interaction of SLP65 with PLCγ2, a known interactor of SLP65 (**Figure 1A**). This confirms that the interaction between SLP65 and GEF-H1 occurs at the endogenous protein level. In addition to this, we also employed proximity ligation assay (PLA) to investigate the interaction between SLP65 and GEF-H1 in unstimulated DG75EB/HA-RhoA cells (**Figure 1B**). Adjacent binding of the antibodies (red dots) used for the assay suggests that the corresponding proteins are localized at a proximity 10-40 nm in B cells (**Figure 1B**). Moreover, this interaction was also observed in primary B cells derived from mouse bone marrow (BM) and spleen as well as human peripheral blood derived B cells (**Figure 1C**). Thus, using three different methods and also B cells from different sources, we could show that GEF-H1 is a novel interacting partner of SLP65. In this context, one relevant question could be the relative expression level of GEF-H1 and SLP65 in different B cells used. To address this, we performed western blot to detect total protein expression of GEF-H1 and SLP65 in primary mouse B cells and DG75EB/HA-RhoA cells (**Figures S1A, B**). DG75EB/HA-RhoA (DG) cells express considerably higher level of GEF-H1 and SLP65 compared to BM derived pre-B cells while the expression is similar to mature (CD43^-^) splenic (SP) B cells (**Figures S1A, B**). Nevertheless, the interaction of SLP65 with GEF-H1 holds true for all sources of B cells analyzed irrespective of the difference in the expression level. Next, to address the functional relevance of this interaction, we checked whether SLP65 activation also leads to activation of GEF-H1. To this end, we used our triple knock out (TKO) cell system with inducible SLP65 (**Figure S1C**). TKO cells are derived from the early pre-B cells from Rag2^-/-^ λ5^-/-^ SLP65^-/-^ mice transduced with an ER^T2^-SLP65 fusion protein (45). When transduced with pre-recombined μHC and λ5 LC, the cells express ectopic pre-BCR (**Figure S1D**) and upon treatment with 4-hydroxy tamoxifen (4-OHT), SLP65 is activated leading to calcium mobilization (**Figure S1E**). We also analyzed the relative level of SLP65 expression in TKO-EST cells compared to primary B cells. As TKO-EST cells overexpress ERT2 fused SLP65 protein, the expression level is higher compared to BM derived pre-B cells but similar to mature splenic B cells (**Figure S1F**). Using this system, we found that SLP65 activation leads to increased phosphorylation of GEF-H1 (**Figure 1D**).

**Table 1:** SLP65 interaction partners as identified by split-ubiquitin yeast-two-hybrid assay.

Protein name	Gene symbol
Polycomb group ring finger 2	PCGF2
Microtubule-associated protein 1B	MAP1B
pyruvate kinase	PKM2
kinesin family member 21B	KIF21B
Kelch repeat and BTB (POZ) domain containing 6	KBTBD6
Polyglutamine binding protein 1	PQBP1
Heat shock protein 90kDa alpha B 1	HSP90AB1
G patch domain containing 3	GPATC3
Trinucleotide repeat containing 18	TNRC18
Rho/rac guanine nucleotide exchange factor GEF-2 (GEF-H1)	ARHGEF2

### SLP65 Regulates RHOA *In Vivo*

**Figure 2:**
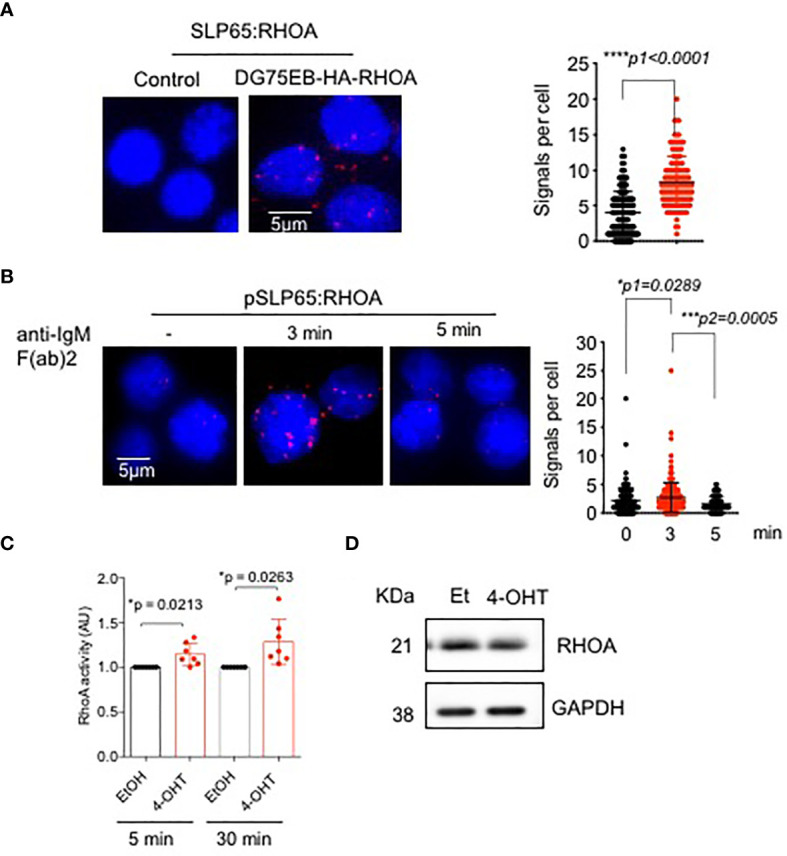
SLP65 interacts with RHOA and regulates its activation. **(A)** Left- PLA showing the association of SLP65 and RHOA in DG75EB/HA-RhoA cell line. Only secondary antibodies are used in the controls. Close proximity is shown as red dots. Right- quantification of number of signals per cell, error bars represent mean ± SD. Unpaired t-test, two-tailed. **(B)** Left- PLA showing the association of RHOA with phosphorylated SLP65 in DG75EB/HA-RhoA cell line upon BCR stimulation. Cells were stimulated with anti-IgM F(ab)_2_ antibody for the specified time period. Right- quantification of number of signals per cell, bars represent mean ± SD. Unpaired t-test, two-sided. **(C)** Detection of total cellular RHOA activity by G-LISA RHOA activation assay. Reconstituted bone marrow derived pre B cells from TKO mice were treated with ethanol (control) or 4-OHT to induce SLP65 for the indicated time point. Equal amounts of cell lysates from control and 4-OHT treated cells were used for the assay. Statistical analysis: one sample t test. **(D)** Western blot analysis for the detection of RHOA upon SLP65 activation in TKO cells. The cells were reconstituted with µ-heavy chain, λ5 and inducible SLP65-ER^T2^ construct and then induced with 2µM 4-OHT or Et for 30 min to activate SLP65. GAPDH is used as loading control. *p<0.05, ***p< 0,001, ****p<0.0001, ns, not significant.

Since GEF-H1 activates RHO GTPases, specifically RHOA, RHOB and RHOC, we asked the question if RHOA is also regulated by SLP65. To this end, we employed PLA to determine the association between SLP65 and RHOA in unstimulated DG75EB/HA-RhoA cells. As shown in **Figure 2A**, red dots indicate close proximity between the two proteins suggesting their interaction. Importantly, stimulating the BCR with anti-IgM F(ab)’2 antibody for 3 min resulted in an increased proximity between phosphorylated SLP65 and RHOA (**Figure 2B**). This interaction was reduced after 5 min indicating that RHOA is recruited to active SLP65 at very early time points. Our data suggests that RHOA can interact with SLP65 in two different modes - one is direct binding, the other is indirect binding through GEF-H1. We also analyzed the relative level of RHOA expression in DG75EB/HA-RhoA cells with that of primary B cells. As depicted in **Figure S1A**, DG75EB/HA-RhoA cells express higher and similar level of RHOA compared to BM derived pre-B cells and splenic B cells respectively. Next, we further investigated whether inducing SLP65 activity results in activating RHOA. Indeed, activating SLP65 in TKO-EST cells using 4-OHT increased the total cellular activity of RHOA (**Figure 2C**) already after 5 min of antibody stimulation without changing its expression level (**Figure 2D**).

Altogether, these data indicate that recruitment of RHOA and GEF-H1 to the SLP65 interactome takes place as an early event and seems to play a role in stimulating the activation of RHOA.

### SLP65 Regulates PTEN Through RHOA

RHOA was previously shown to activate PTEN function (33, 34), and may therefore be involved in inhibiting PI3K activity in pre-B cells. Thus, we hypothesized that GEF-H1/RHOA may provide the molecular platform that mediates SLP65 signals through PI3K pathway.

**Figure 3:**
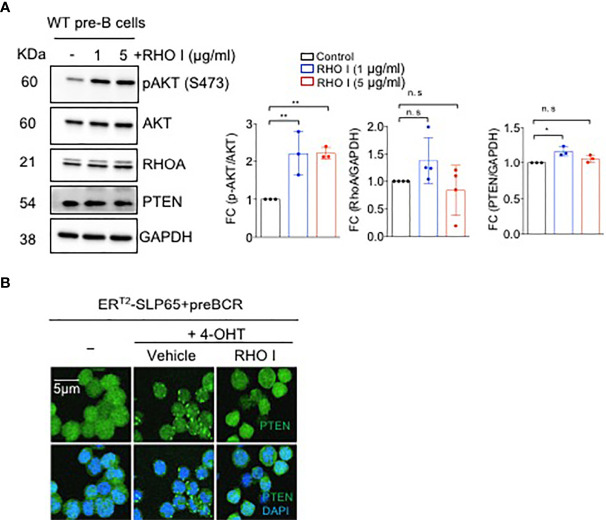
SLP65 regulates PTEN localization through RHOA. **(A)** Left- Wild type (WT) bone marrow derived pre-B cells were treated with RHO-specific inhibitor C3-toxin (RHO I) for 16 hours or were left untreated for 16 hrs. Total cell lysates were prepared and immunoblotting was performed. Data are representative of 3 independent experiments. Right- quantification of the western blots. RhoA and PTEN blots are normalized with GAPDH while p-AKT is normalized with total AKT. Bars represent mean ± SD. Statistical test- one-way ANOVA with multiple comparison test. **(B)** SLP65 deficient pre-B cells were retrovirally reconstituted with ERT2-SLP65 and were induced with 4-OHT for 30 min with or without RHO (I) Cells treated with vehicle were used as a control. Cells were then fixed with 2% paraformaldehyde and labeled with DyLight 488 conjugated anti-PTEN antibody and DAPI staining for immunofluorescent microscopy. Images were captured by using a Zeiss LSM 780 laser scanning confocal microscope with GaAsP detectors and visualized with Imaris software. *p<0.05, **p<0.01, ns, not significant.

To investigate the link between PTEN/PI3K and SLP65-dependent RHOA activation, we first studied the effect of RHOA inhibition on PI3K pathway. Interestingly, treatment of pre-B cells with a RHO inhibitor significantly enhanced AKT activation as shown by increased serine 473 (S473) phosphorylation and a small but significant elevation of PTEN expression (**Figure 3A**). Next, we monitored the intracellular localization of PTEN upon induction of SLP65 function in our inducible TKO-EST cell system (**Figure S1C**). SLP65-induction led to PTEN activation and membrane localization (**Figure 3B**). In addition, treating the cells with RHO inhibitor prevented PTEN clustering and subsequent recruitment towards the membrane (**Figure 3B**). We also checked if RHO inhibition changes PTEN expression or AKT activation in these cells. PTEN total protein expression was unchanged upon RHO inhibition (**Figures S2A, B**). This could be explained by the fact that upon RHO inhibition, the subcellular localization of PTEN changes without altering the total protein amount. However, for AKT, we see a strong increase in AKT phosphorylation in TKO-EST cells upon SLP65 activation, which was reduced upon RHO inhibition but still remain higher compared to the control untreated cells (**Figures S2A, B**). This was similar to what we see in the primary WT bone marrow derived B cells.

These data indicate that activation of RHOA by SLP65 leads to induction of the RHOA/PTEN pathway thereby counteracting PI3K signaling.

### RHOA Is Indispensable for Early B Cell Development

**Figure 4:**
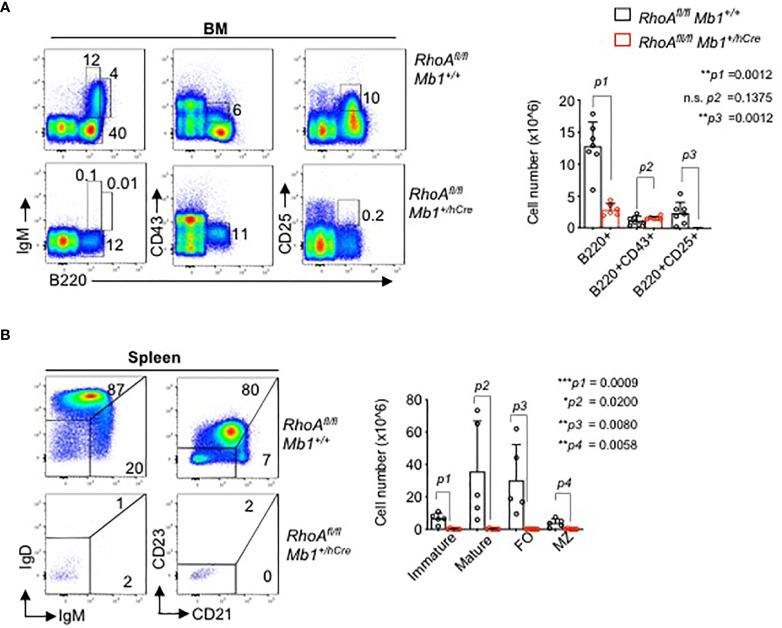
B cell specific *RhoA* deletion leads to developmental block at the pro B cell stage. **(A)** Flow cytometric analysis of freshly isolated cells from bone marrow (BM) of *RhoA^fl/fl^ and RhoA^fl/fl^ Mb1^+/hCre^* mice for surface expression of the indicated markers. Data are representative of 6 mice. Right: absolute cell numbers of B220^+^, pro-B cells (B220^+^CD43^+^), and pre-B cells (B220^+^CD43^-^) in the BM. **(B)** Flow cytometric analysis of cells isolated from spleen of *RhoA^fl/fl^ and RhoA^fl/fl^ Mb1^+/hCre^* mice. Right: absolute cell numbers of splenic B cells, immature (IgM^hi^IgD.), mature (IgM^low^IgD^+^) MZ: marginal zone (CD21^hi^CD23^-^), FO: follicular (CD21^low^CD23^+^). N=5 mice, statistical significance was calculated using two-tailed unpaired t-test. *p<0.05, **p<0.01, ***p< 0,001, ns, not significant.

Next, to analyze the effects of RHOA in B cell development *in vivo*, we generated B cell specific *RhoA* deleted mouse line. Mice harboring a “floxed” *RhoA* allele (*RhoA^fl/fl^*) were crossed to *Mb1^+/hCre^* transgenic mice to enable *RhoA* gene deletion at Pro-B cell stage. The animals showed a complete block in B cells development at the pro-B cell stage as indicated by the absence of B220^+^CD25^+^ pre-B cells in bone marrow (**Figure 4A**) and absence of mature B cells in the spleen (**Figure 4B**). These data suggest that RHOA plays an indispensable role in pre-B cell differentiation. This phenotype resembles the phenotype of *Pten^fl/fl^* mice (46) and *FoxO1^fl/fl^* mice (14) crossed to *Mb1^+/hCre^*, which is in line with our hypothesis that RHOA mediates SLP65 function in pre-B cell differentiation through suppressing PI3K signaling (47).

**Figure 5:**
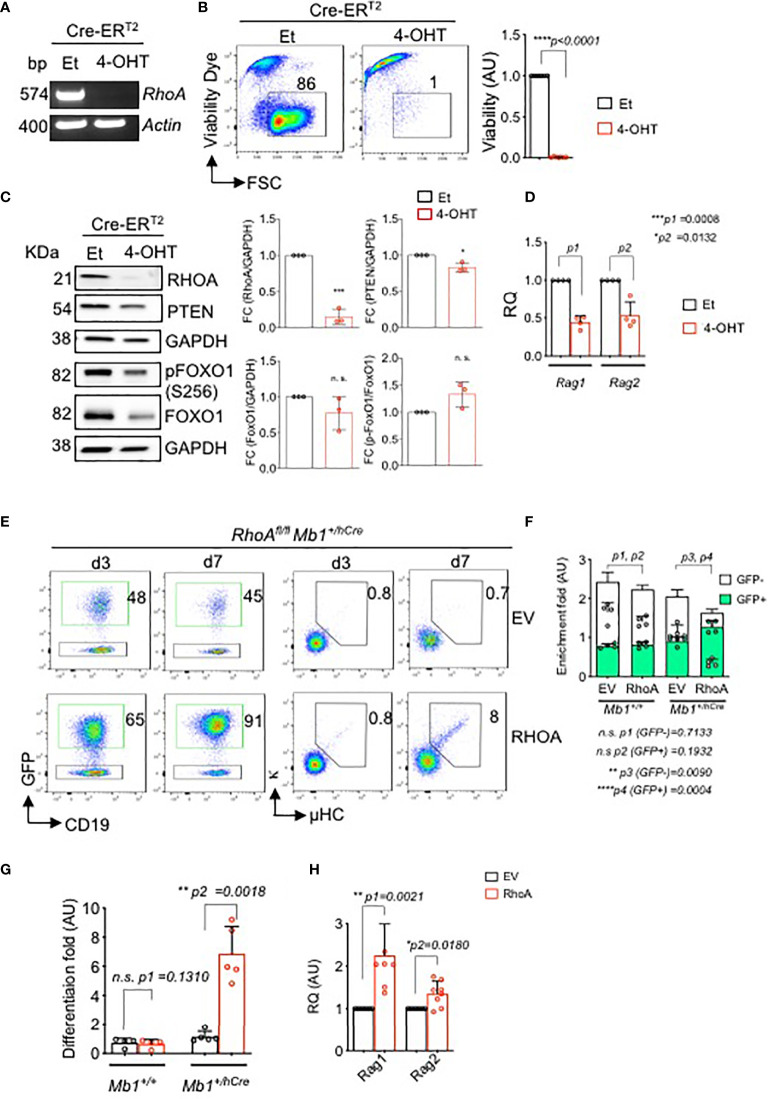
*In vitro* deletion of *RhoA* leads to cell death and impaired immunoglobulin gene rearrangement and B cell differentiation. *RhoA^fl/fl^ Mb1^+/+^* pre-B cells were transduced with Cre-ER^T2^. Cells were then treated with either 4-OHT to induce Cre expression, or with Et. **(A)** RT-PCR analysis for *RhoA* gene expression after 2 days of tamoxifen induction. *Actin* was used as a loading control. bp: base pair. **(B)** Left- flow cytometric analysis of viability of RHOA deleted cells using Sytox dye after 4 days of tamoxifen induction. Right- the fold change of living cells after treatment with either Et or 4-OHT at day 4. N=6 independent samples per group, and error bars represent mean ± SD. Paired t-test, two-tailed. **(C)** Left- western blot analysis showing expression of RHOA, PTEN and phospho FOXO1 after 2 days of tamoxifen induction. Data representative of three independent experiments. Right- quantification of the western blots. RHOA, PTEN and total FOXO1 blots are normalized with GAPDH while p-FOXO1 is normalized with total FOXO1. Bars represent mean ± SD. Statistical test- unpaired t test. **(D)** Quantitative RT-PCR showing *Rag1* and *Rag2* expression after *RhoA* deletion. N=4 independent samples per group, and error bars represent mean ± SD. Paired t-test, two-tailed. **(E)** *RhoA^fl/fl^ Mb1^+/+^* or *RhoA^fl/fl^ Mb1^+/hCre^* pre-B cells were reconstituted with either an empty vector (EV, pMIG) or with *RhoA* retroviral expression vector (pMIG-RHOA). Left- Flow cytometric analysis showing enrichment of *RhoA^fl/fl^ Mb1^+/hCre^* cells transduced with either an EV or RHOA (GFP^+^ population) after 3 and 7 days of transduction. Right- Flow cytometric analysis showing an increase in the percentage of differentiated cells (µ^+^kappa^+^) in *RhoA*-deficient cells which were reconstituted with *RhoA* (N=5). **(F)** Quantification of enrichment of GFP^+^ pre-B cells from *RhoA^fl/fl^ Mb1^+/+^* or *RhoA^fl/fl^ Mb1^+/hCre^* mice reconstituted with either EV or *RhoA*. Enrichment fold represents the ratio of CD19^+^GFP^+^ or CD19^+^GFP^-^ at day 7 relative to day 3. N=4 independent samples per group, and error bars represent mean ± SD. Paired t-test, two-tailed. **(G)** Quantification of enrichment of differentiated cells (µ^+^kappa^+^) as calculated by the ratio of µ^+^kappa^+^ at day 7 relative to day 3. N=4 independent samples per group, and error bars represent mean ± SD. Paired t-test, two-tailed. **(H)** Quantitative RT-PCR showing up-regulation of *Rag1* and *Rag2* gene after *RHOA* reconstitution in *RhoA^fl/fl^ Mb1^+/hCre^*. N=4 independent samples per group, and error bars represent mean ± SD. Paired t-test, two-tailed. *p<0.05, **p<0.01, ***p< 0,001, ****p<0.0001, ns, not significant.

To confirm this observation *in vitro*, we introduced a 4-OHT-inducible Cre (Cre-ER^T2^) retroviral vector, or a control ER^T2^, into BM-derived *RhoA^fl/fl^* pre-B cells. Inducible deletion of the *RhoA* gene led to rapid cell death within 96 hours (**Figures 5A, B**, **Figures S3A, B**). Interestingly, RHOA knock out shows a small but significant reduction of PTEN expression and a slightly reduced expression of total FOXO1 (**Figure 5C** and **Figure S3C**). However, the relative level of phosphorylated FOXO1 is increased in the RHOA deleted cells indicating a reduced activity of this transcription factor (**Figure 5C** and **Figure S3C**). This suggests that RHOA expression is required for proper PTEN expression in pre-B cells and RHOA deficiency interferes with the regulation of PI3K signaling possibly through a negative feedback mechanism by reducing PTEN expression. Of note, the protein expressions were determined 48 hours after *RhoA* deletion and no cell death was evident at this time point. Thus, the changes in protein expression are not merely due to loss of cells but due to loss of *RhoA*. In addition, *RhoA*-deficient pre-B cells show significant decrease in *Rag1* and *Rag2* gene expression levels (**Figure 5D** and **Figure S3D**) indicating that they are unable to undergo LC gene recombination. This could be explained by the reduced activation of FOXO1 as demonstrated above. To confirm this observation, BM-derived pre-B cells from either *RhoA^fl/fl^* or *RhoA^fl/fl^* Mb1^+/hCre^ mice were reconstituted with pMIG-RHOA retroviral vector or with an empty vector (EV, pMIG) (**Figure 5E** and **Figure S3E**). Whereas ectopically expressed RHOA (GFP+) was not enriched in *RhoA^fl/fl^* pre-B cells (**Figure S3E**), RHOA^pos^ population was significantly enriched in RHOA-deficient pre-B cells and also led to increased cell viability (**Figures 5E, F**). Importantly, RHOA overexpression restored the ability of these cells to differentiate as represented by the elevated percentage of cells expressing Igκ (**Figures 5E, G**) and by increased levels of *Rag1* and *Rag2* gene expression (**Figure 5H** and **Figure S3F**). Next, we investigated whether ectopic expression of either FOXO1 or PTEN can overcome the differentiation block observed in *RhoA*-deficient pre-B cells. Interestingly, neither of these factors could compensate for RHOA deficiency in driving pre-B cell differentiation (**Figure S3G**). Altogether, these data explain the developmental block observed *in vivo* and suggest that RHOA is an important factor for *Ig* gene rearrangement acting downstream of FOXO1/PTEN pathway.

### RHOA Is Required for the Survival of B Cell Precursor Acute Lymphoblastic Leukemia (BCP-ALL)

**Figure 6:**
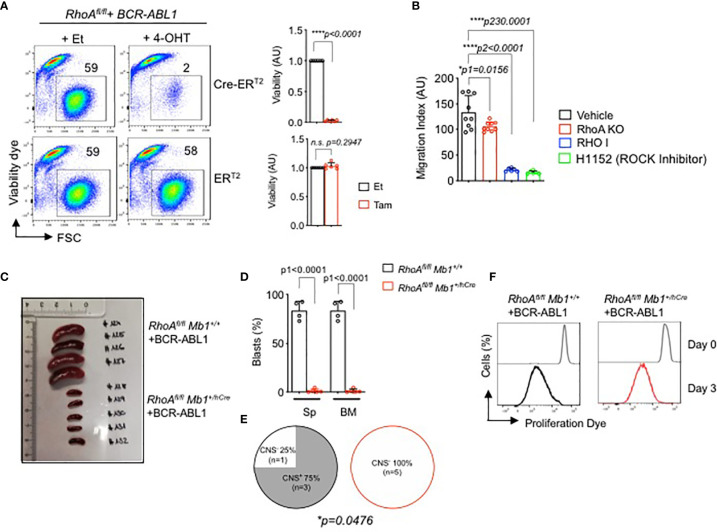
RHOA is required for BCR-ABL1-induced pre-B cell transformation. **(A)** *RhoA^fl/fl^* pre-B cells were transformed with BCR-ABL1 and were then transduced with Cre-ER^T2^. Cells were treated with either 4-OHT to induce Cre expression, or with Et. Left- The percentage of living cells were determined by flow cytometry using Sytox as an excluding dead cell stain. Right- quantification of the percentage of living cells after 3 days of tamoxifen induction relative to control cell. N=6 independent samples per group, and error bars represent mean ± SD. AU: arbitrary unit. **(B)** *RhoA^fl/fl^*-BCR-ABL1 transformed cells were treated with RHO inhibitor (RHO I, 2µg/ml), H1152 (ROCK inhibitor H1152, 5µM) or with vehicle for 16 hours, then were subjected to migration gradient toward CXCL12 (100ng/ml) for 16 hours*. RhoA^fl/fl^ Mb1^+/hCre^* -BCR-ABL1^+^ cells were also used for migration assay. The cells that migrated in the lower chamber were counted by trypan blue. **(C, D)**. *RhoA^fl/fl^* and *RhoA^fl/fl^ Mb1^+/hCre^* pre-B cells were transformed with BCR-ABL1 and were then injected intravenously into immunodeficient RAG2^-/-^ γc^-/-^ mice. The mice were sacrificed after three weeks when the control mice showed leukemic symptoms. **(C)** Spleen (Sp) size. **(D)** Quantification of total number of BCR-ABL1+ cells in the Sp and bone marrow (BM). **(E)** CNS-infiltration as assessed by semi-quantitative scoring **(D, E)**. Statistical significance was determined using unpaired t-test, two-tailed. **(F)** *In vitro* proliferation of the *RhoA*-deficient transformed cells recovered from the bone marrow of the RAG2^-/-^ γc^-/-^ mice **(C, D)**. The cells were cultured for 1 week and labelled with a proliferation dye. Cells were analyzed by flow cytometry directly after the labelling (Day 0) and after 3 days (Day 3). One representative experiment is shown. *RhoA^fl/fl^ Mb1^+/+^* (N=4), *RhoA^fl/fl^ Mb1^+/hCre^* (N=5). *p<0.05, ****p<0.0001, ns, not significant.

Since RHOA is indispensable for pre-B cell development, we investigated if it has any role in the development of pre-B cell malignancies such as in BCP-ALL. Here, we generated BCR-ABL1 transformed bone marrow derived pre-B cells from *RhoA^fl/fl^* mice. For inducible deletion of the *RhoA gene*, we introduced a 4-OHT-inducible Cre-ER^T2^ vector into the BCR-ABL1-transformed *RhoA^fl/fl^* cells. Inducible deletion of the *RhoA* gene led to cell death of the BCR-ABL1-transformed pre-B cells (**Figure 6A**). Interestingly small GTPases, like RHOA, often function downstream of G-protein coupled receptors, such as CXC family chemokine receptor 4 (CXCR4) or integrins, which are important co-receptors regulating B cell development and migration (28). Previously, we showed that the signaling pathways of IL7R and CXCR4 are tightly regulated by the activity of the oncogenic kinase BCR-ABL1 (48). Here we show that the inhibition or deletion of RHOA significantly reduced cell migration toward CXCL12, the ligand of the chemokine receptor CXCR4 (**Figure 6B**).

Next, we studied whether *RhoA* is also required for the proliferation and survival of transformed B cells *in vivo*. Since BCR-ABL1 transformed pre-B cells die after *RhoA* deletion *in vitro* (**Figure 6A**), we isolated bone marrow derived pro- B cells from *RhoA^fl/fl^* Mb1^+/hCre^ mice, transformed them with BCR-ABL1 and injected them intravenously into immunodeficient Bl6 *Rag2 ^-/-^ γc ^-/-^* mice. *RhoA^fl/fl^* Mb1^+/+^ pro-B cells transformed with BCR-ABL1 were used as control group. The mice were then sacrificed after two weeks when the control mice showed leukemia symptoms such as hind limb paralysis. Mice injected with *RhoA*-deficient BCR-ABL1 cells did not develop leukemia when compared to mice injected with control cells as evidenced by reduced spleen size and total number of blasts in both spleen and bone marrow (**Figures 6C, D**). Consequently, significantly less CNS infiltration was observed for mice injected with *RhoA*-deficient BCR-ABL1 cells compared to mice injected with control *RhoA*-proficient BCR-ABL1 cells (**Figure 6E**) (49). In order to exclude that *RhoA* deficient BCR-ABL1 transformed cells failed to engraft *in vivo*, the bone marrow from both mouse groups was recovered and cultured *in vitro* in medium without IL7 which enables the growth of transformed cells only. After one week, a pure population of transformed cells was generated from both groups (*RhoA*-competent and *RhoA*-deficient) indicating that all mice were successfully engrafted with BCR-ABL cells. Next, we performed an *in vitro* proliferation assay with recovered BCR-ABL cells. Cells were labelled with fluorescent cell proliferation dye and the rate of proliferation was measured by flow cytometry after 3 days. *RhoA* deficient transformed cells were shown to proliferate to a similar extent as transformed cells with intact *RhoA* (**Figure 6F**). These data suggest that *RhoA* deficiency does not affect the proliferation of the transformed cells *in vitro*. Nevertheless, *RhoA* deficiency prevents leukemia development and disease progression *in vivo* (**Figures 6C–E**).

### RHOA Is Required for the Development of Marginal Zone B Cells

**Figure 7:**
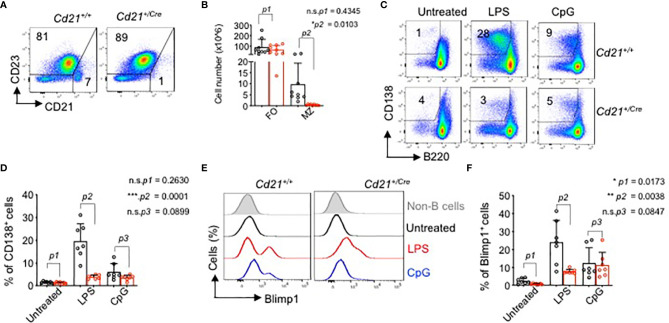
Impaired marginal zone B cell development and plasma cell differentiation in *RhoA^fl/fl^ Cd21^+/Cre^ mice.* ***(*A*)*** Flow cytometric analysis of splenic cells from *RhoA^fl/fl^ and RhoA^fl/fl^ Cd21^+/hCre^* mice for surface expression of the indicated markers. Data are representative of 9 mice. **(B)** Absolute cell numbers of MZ (CD21^hi^CD23^-^) and FO (CD21^low^CD23^+^) cells. Statistical analysis- two tailed unpaired student t test. **(C)** Flow cytometric analysis of plasma cell differentiation (CD138^+^) of splenic cells from *RhoA^fl/fl^* and *RhoA^fl/fl^ Cd21* ^+/Cre^ mice upon stimulation with 2.5 µg/ml LPS or 2.5 µM CpG after 4 days of treatment. Cells were pre-gated on CD19^+^ cells. **(D)** Quantification of CD138^+^ B cells in LPS-, CpG- and non-treated splenocytes at day 4 post stimulation. N=7 independent mice per group, and error bars represent mean ± SD. Unpaired t-test, two-tailed. **(E)** Histogram showing the expression of BLIMP-1 in B cells from *RhoA^fl/fl^* and *RhoA^fl/fl^ Cd21* ^+/Cre^ mice upon stimulation with LPS and CpG as detected by Intracellular flow cytometry. **(F)** Quantification of BLIMP-1^+^ splenic B cells (pre-gated on CD19^+^ cells). N=7 independent mice per group, and error bars represent mean ± SD. Unpaired t-test, two-tailed. *p<0.05, **p<0.01, ***p< 0,001, ns, not significant.

To better understand the role of RHOA in late B cell developmental stages, we crossed the *RhoA^fl/fl^* mice to *Cd21* ^+/Cre^ transgenic mice (50). CD21 is expressed at the time when immature/transitional B cells differentiate into mature long-lived peripheral B cells (51). Results show that B cell development in the bone marrow was not affected in *RhoA^fl/fl^ Cd21* ^+/Cre^ mice (**Figure S4A**). A strong reduction in *RhoA* gene expression and a concomitant increase in *RhoB* and *RhoC* genes were observed in the splenic B cells from *RhoA^fl/fl^ Cd21* ^+/Cre^ mice (**Figure S4B**) indicating a compensatory mechanism in the cells. Interestingly, *Rock1* and *Rock2*, the two RHO activated kinases, are reduced in the *RhoA^fl/fl^ Cd21* ^+/Cre^ mice (**Figure S4B**). In the spleen, although there were no significant changes in the follicular (FO) B cells percentages or total cell number (**Figures 7A, B**), the marginal zone (MZ) B cell population was strongly reduced in *RhoA-*deficient mice compared to control mice (**Figures 7A, B** and **Figure S4C**). Thus, we investigated whether this phenotype was correlated with altered functional responses. We stimulated splenic B cells from *RhoA^fl/fl^ Cd21* ^+/Cre^ or *RhoA^fl/fl^ Cd21* ^+/+^ mice with anti-BCR (anti-µHC), CpG, LPS and anti-CD40 antibody and measured the surface expression of early activation markers CD86 and CD69 after 24 hours of stimulation. (**Figure S4D**). *RhoA-*deficient B cells showed some reduction in CD86 surface levels, but not CD69, in response to the stimulation in comparison to wildtype B cells (**Figures S4D–F**). Nevertheless, in response to LPS stimulation, *RhoA-*deficient B cells showed impaired ability to proliferate and become CD138^+^/B220^lo^ plasma cells (**Figures 7C, D**) and expressed lower levels of BLIMP-1 (**Figures 7E, F**) after 4 days of stimulation. Interestingly, we found that under stimulation with CpG, *RhoA-*deficient B cell proliferated in similar pattern as control cells (**Figures 7C, D**). These results suggest that while RHOA may be dispensable for proliferation through TLR9, it is absolutely required for TLR4. The reduced expression of BLIMP-1 suggests that *RhoA-*deficient B cells cannot be committed into terminal differentiation, most likely due to deregulated PI3K signaling (19). Altogether, the data suggest that functional RHOA is required for proper selection and regulation of peripheral B cells.

### Impaired Immune Response in *RhoA* Deleted Mice

Next, we measured the serum level of IgM and IgG antibodies in *RhoA^fl/fl^ Cd21* ^+/Cre^ mice compared to the control group. Serum IgM did not show any alteration (**Figure S5A**) while IgG titre was reduced in *RhoA^fl/fl^ Cd21* ^+/Cre^ mice specifically at the older age (**Figure S5A**). Importantly, *RhoA-*deficient mice had an increased amount of autoreactive anti-double stranded DNA (dsDNA) antibodies in sera compared to control mice (**Figure S5A**). Thus we investigated whether *RhoA^fl/fl^ Cd21* ^+/Cre^ mice develop autoimmune diseases such as kidney failure with age. Histological analysis of HE and PAS stained kidney sections revealed no pathology of glomerulonephritis (**Figure S5B**). In addition, proteinuria levels were similar in both groups (**Figure S5C**) indicating no autoimmune phenotype in these mice.

**Figure 8:**
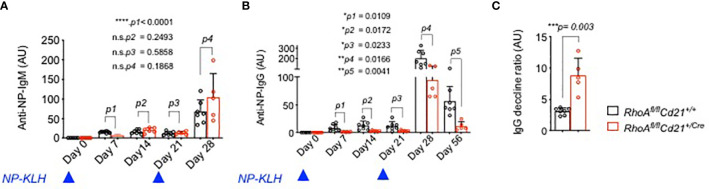
Impaired humoral immune response of *RhoA^fl/fl^ Cd21^+/Cre^ mice.* ***(*A*)*** Six-eight weeks old *RhoA^fl/fl^* (N=7) and *RhoA^fl/fl^ Cd21^+/Cre^* (N=8) mice were immunized with 100µg NP(24)KLH + 100µg CpG-ODN1826 in PBS or with 100µg CpG-ODN1826 (control). Serum level of NP specific IgM **(A)** and IgG **(B)** antibodies were measured by ELISA at the specified days after first immunization (day 0) and booster on day 21. No NP-specific antibodies were detected in control mice injected with adjuvant (CpG-ODN1826) hence excluded from the figure. Statistical analysis- One-way ANOVA. **(C)** The decline of anti-NP-IgG titers was calculated as the ratio of IgG titers at day 28 relative to day 56. Statistical significance was determined using unpaired t-test, two-tailed. *p<0.05, **p<0.01, ***p< 0,001, ****p<0.0001, ns- not significant.

To assess the ability of *RhoA*-deficient B cells to undergo GC reaction and mount immune responses, we immunized *RhoA^fl/fl^ Cd21* ^+/Cre^ and *RhoA^fl/fl^ Cd21* ^+/+^ control mice with 4-hydroxy-3-nitrophenylacetyl (24)-keyhole limpet hemocyanin (NP(24)KLH) and analyzed the serum titre of NP specific IgM and IgG antibodies. Although IgM did not show an alteration (except for a reduction at day 7, **Figure 8A**), *RhoA^fl/fl^ Cd21* ^+/Cre^ mice showed significantly reduced IgG titres at days 7,14 and 21 after the first immunization compared to control mice (**Figure 8B**). After a second injection at day 21, NP specific IgG titer increased in *RhoA^fl/fl^ Cd21* ^+/Cre^ mice at day 28 but to a lesser extent than control mice (**Figure 8B**). Also, it declines at a much faster rate in *RhoA^fl/fl^ Cd21* ^+/Cre^ than control mice (**Figure 8C**) indicating that *RhoA*-deficient mice fail to mount long-lived humoral immunity.

### Role of RHOA in Mature B Cell Malignancy

So far, we showed that RHOA interacts with SLP65 and is directly involved in B cell differentiation at earlier as well as later developmental stages. RHOA has been shown to promote the reorganization of the actin cytoskeleton thereby regulating cell shape, attachment, and motility (22). It has previously shown that a constitutive activity of the BCR as well as altered cytoskeleton structure is associated with B cell-derived neoplasms, such as the activated B cell subtype of diffuse large B cell lymphoma (DLBCL, ABC) or chronic lymphocytic leukemia (CLL) (52). Interestingly, increased levels of RHOA were reported for CD5^+^ B cells (53) and therefore we hypothesized that RHOA may play a role in the development and survival of CLL B cells.

To check this, we studied the effect of *RhoA* deletion in DG75EB/HA-RhoA cell line by inactivating the *RhoA* gene using CRISPR/Cas9 genome editing (**Figures S6A, B**). Induction of *RhoA* deletion in these cells (**Figure S6C**) by removing doxycycline from the culture media resulted in an increased cell size (**Figure S6D**). Interestingly, RhoA deletion resulted in up-regulation of RAC1 and RAC2 indicating that the expression of RAC1/2 proteins is regulated by the amount of RHOA in the cells (**Figure S6C**).

**Figure 9:**
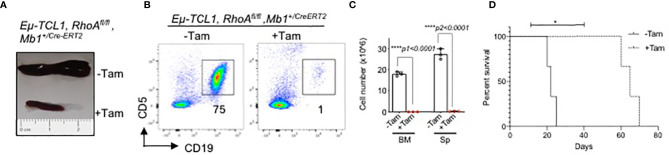
Rapid elimination of CLL B cells after inducible deletion of *RhoA*. **(A)** Representative picture of spleen size of diseased mice treated with or without Tamoxifen. **(B)** Flow cytometric analysis showing elimination of CLL B cells (CD19^+^CD5^low^) after 3 weeks of tamoxifen induction. **(C)** Absolute number of splenic CLL B cells. N=3 mice per group, unpaired t-test, two-tailed. **(D)** Kaplan-Meier survival curve showing the survival of Eµ-TCL1 RhoA^fl/fl^ Mb1^+/CreERT2^ mice after Tamoxifen-induced RhoA deletion (+Tam) or without treatment (-Tam). Results from 3 individual mice are indicated (n=3). The P-value was determined by Mantel-Cox log-rank test (*p < 0.05, ****p<0.0001).

Next, we studied the effect of inducible *RhoA* deletion in CLL mouse model. *Eμ-TCL1* mice (54) were crossed with Mb1^+/Cre-ERT2^ strain (55) which expresses a tamoxifen-inducible form of the *Cre* recombinase under the control of the *Mb1* promoter region. Then, the mice were crossed with *RhoA^fl/fl^* mice to generate *Eµ-TCL1 RhoA^fl/fl^ Mb1^+/Cre-ERT2^* strain. The mice were maintained for 10 months until they developed the disease which is characterized by accumulation of CLL B cells (CD19^+^CD5^low^). After 3 weeks of tamoxifen-induced *RhoA* deletion, TCL1-transgenic mice had significantly smaller spleens as compared to untreated mice (**Figure 9A**). In addition to this, *RhoA* deletion also led to the elimination of CLL-B cells in spleen and bone marrow (**Figures 9B, C**) and significantly improved the survival rate of the mice (**Figure 9D**). This suggests that RHOA is essential for the development and maintenance of CLL-B cells.

## Discussion

SLP65 regulates pre-BCR/BCR signaling cascade which is required to generate healthy and functional B cells (13). It is well known that PI3K is a key player in *Ig* genes rearrangement, as well as for subsequent differentiation, proliferation and selection processes (56). Regulating PI3K signaling in B cells is of central importance to understand B cell survival and differentiation. In this study we have revealed a novel mechanism connecting SLP65 and PI3K through small GTPases. Particularly, by using an inducible ER^T2^-SLP65 experimental system and PLA assays, we have identified RHOA as a novel interacting protein of active SLP65.

The lipid phosphatase PTEN is known as a negative regulator of PI3K signaling. It was previously shown that RHOA/ROCK pathway can control PI3K/AKT signaling *via* the regulation of intracellular localization of PTEN in leukocytes and human embryonic kidney cells (33, 34), which influences chemotaxis. Here we show that activation of RHOA by SLP65 leads to induction of this RHOA/PTEN pathway thereby counteracting PI3K signaling. The physical interaction between SLP65 and GEF-H1 offers RHOA to be the key signaling molecule that links SLP65 activation and PI3K pathways down-regulation.

This was further confirmed by *in vivo* data showing an indispensable role of *RhoA* at early B cell developmental stages. Similarly, *RhoA* reconstitution *in vitro* has recovered *Ig* recombination process in *RhoA-deficient* pro-B cells and enhanced pre-B cell differentiation. *Ig* rearrangement is highly regulated by PI3K, and genetic ablations of either PTEN or the PI3K downstream effector FOXO1 in B cells lead to an early developmental blockage (13–15, 46). Deletion of *RhoA* by *CD19Cre/+* leads to decrease expression of BAFF receptor and a loss of BAFF-mediated AKT activation resulting in impaired splenic B cell development (28). On the other hand, deletion of RHOA in hematopoietic stem cells using Mx-Cre led to complete block in B cell development in the bone marrow with increased number of CLP (28). This indicates that RHOA is required for B cell progenitor/precursor differentiation. Our *Mb1^+/hCre^* mouse model provided an advantage over previously described *Cd19*-Cre transgenic model as it enables *RhoA* deletion even at an early developmental stage (pro-pre B cell).

On the other hand, deletion of *RhoA* at later developmental stage, using *Cd21* ^+/Cre^ mouse model, led to a reduction in the total number of splenic B cells and the complete absence of MZ B cells. Mice deficient for *Cdc42* (57) or *Rac1* (58) were shown to have decreased numbers of FO and MZ B cells whereas *Rac2*-defcient mice showed a reduction only in MZ B cells (58). On molecular signaling level, the *Cdc42*-deficient B cells also show decreased AKT activation, impaired B cell proliferation and compromised antibody response (30).

MZ B cells express polyreactive BCRs as well as TLRs and are thought to have a lower threshold than conventional FO B cells, which enable them to initiate early low-affinity antibody responses. The significant reduction of MZ B cells in *RhoA-deficient* mice may explain the impaired activation in response to LPS stimulation and the subsequent differentiation into plasma cells *in vitro* and the significant decrease in total IgM and IgG especially within the first week post immunization. The reduction in the generation of CD138^+^ B cells could be due to a reduced survival signals. Indeed, *RhoA* deficiency was shown to completely block BAFF-mediated AKT activation (28). However, the expression of activation markers CD86 and CD69 in response to different stimuli was similar in WT and *RhoA^-/-^* B cells, this could be due to compensation or functional redundancy by other Rho GTPase family members such as *Cdc42*, *Rac1/2*, *RhoB* and *RhoC* (28). Further studies are necessary to see if other RHO family members also participate in the SLP65/PTEN pathway to regulate B cell differentiation and function.

In mature B cells, deregulated PI3K signaling seems to be important for GC reaction and the generation of high affinity antibodies of IgG types. Previous work has shown a role for one of the Rho-GEFs, ARHGEF1, in the retention of B cells within GC (59). B cell positioning within the GC depends on the interaction with the microenvironment. We found that *RhoA-deficient* B cells showed impaired migration toward CXCL12, the disruption of this signaling pathway could destroy GC architecture, B cell differentiation, and the humoral responses. In the same context BAFF-R-mutant A/WySnJ mice show defective immune response, where the B cells lose active migration toward CCL21, CXCL12, and CXCL13 (60). The cell migration process in B cells seems to strictly depend on RHOA activation. In line with these results, the lack of ROCK2, a serine-threonine kinase acting downstream RHOA, was associated with defective GC polarization (35). Particularly, ROCK2 blocked AKT activation and promoted the transcriptional activity of FOXO1 (35). Interestingly the requirement for DOCK8 to organize a normally polarized B cell immunological synapse is very similar to defects in B cells caused by *Rac2* deficiency, where DOCK8 mutant B cells are unable to form marginal zone B cell and fail to produce long-lasting immunity (61).

Signaling downstream of GTPases were shown to play critical roles in regulating tumorigenesis and metastasis (62). Here we investigated RHOA in two different malignant B cell models CLL and ALL. In both cases, RHOA expression was required for tumor development and survival in both *in vitro* and *in vivo*. Traditionally, overactivation of the Rho GTPase pathways has been associated with malignancy, therefore, Rho/ROCK pathway inhibitors resulted in specific growth inhibition of CML cell line transformation (63). Our results also show that deletion of RHOA leads to amelioration of growth of the malignant B cells indicating RHOA inhibitors may have significant therapeutic relevance for ALL and CLL.

## Materials and Methods

### Cell Culture

Freshly isolated bone marrow cells from WT, RhoA*^fl/fl^* and RhoA*^fl/fl^* mb1 +/hcre mice were cultured in Iscove’s medium (Biochrom AG) containing 10% heat-inactivated fetal calf serum (FCS; Sigma-Aldrich) while triple knock out (TKO) pro B cell line reconstituted with an expression vector for the pre-BCR and inducible form of SLP-65 cells were cultured in Iscove’s medium containing 5% heat-inactivated FCS. The media were supplemented with a supernatant of J558L plasmacytoma cells stably transfected with a vector encoding murine IL7. Phoenix cells and mature splenic cells were cultured in Iscove’s medium containing 10% heat-inactivated FCS without IL-7. All the above-mentioned media were supplemented with 100 U/ml penicillin (Gibco), 2mM L-glutamine, 100 U/ml streptomycin (Gibco), and 50 μM 2-mercaptoethanol (Gibco).

DG75EB/HA-RhoA cells are derived from human Burkitt lymphoma cell line DG75 (64). The cells were stably transfected to express the murine cationic amino acid transporter 1 (*SLC7A1*) to make them susceptible for infection with MMLV-based retrovirus particles (65). These modified cells are termed as DG75EB. To obtain HA-RhoA-expressing DG75EB cells, we retrovirally infected the cells with pRetroX-OneTet-Pruo (Clontech) encoding HA tagged RHOA. Upon treatment with Doxycycline, the cells express HA-RHOA. These cells were cultured in RPMI medium containing 10% heat-inactivated FCS (PAN Biotech) and 1 mg/ml Doxycycline (Sigma Aldrich).

### CRISPR/Cas–Based Genome Editing to Generate Inducible RhoA Knock Out DG75EB Cells

For cloning of CRISPR/Cas-based genome editing constructs, the pSpCas9(BB)-2A-GFP vector was used (Addgene Cataloug number 48138). Design of guide RNA was performed by using the CRISPR/Cas Design software (http://crispr.mit.edu/) against human *RHOA* Exon2, (sgRNA: GAA CTA TGT GGC AGA TAT CGA GG, Score: 85). Oligos for cloning were: 5’-CAC CGG AAC TAT GTG GCA GAT ATC G-3’ and 5’-AAA CCG ATA TCT GCC ACA TAG TTC C-3’. EcoRV was used for activity screening on genomic DNA of transfected cells. Primer sequences used to construct HA-*RHOA* were: forward 5’- AAT TAC GTT GCT GAC ATA GAA GTG GAT GGA AAG CAG GTA GAG TTG GCT-3’ and reverse 5’- CCA TCC ACT TCT ATG TCA GCA ACG TAA TTC TCA AAC ACT GTG GGC ACA TAC ACC TC-3’.

For our study, we used cells cultured in presence of doxycycline to have intact *RhoA* expression (referred to as: DG75EB/HA-RhoA). For experiment where we induced *RhoA* deletion by removal of Dox, the cells are referred to as: DG75EB/HA-RhoA/RhoA-KO.

### Plasmids and Retroviral Transduction

Full-length cDNA encoding murine *RhoA* was subcloned into the retroviral vector backbone of pMIG to generate a RhoA-IRES-GFP expression vector. Plasmids for the expression of *Foxo1*, *Pten* and tamoxifen-inducible form of Cre (*Cre-ERT2*) are described previously (13, 46). Viral supernatants were generated using the Phoenix retroviral producer cell line as described in the manufacturer’s instructions. In summary Phoenix, cells were cultured in Iscove’s culture medium + 10% FCS. Cells were plated at a density of 0.25*10^6^ cells/mL to generate supernatants by using the transfection reagent GeneJuice (Merck Millipore). Retroviral supernatants were harvested after 48 hr. For the subsequent transduction, the respective cells were mixed with supernatants and centrifuged for 3 hours at 300g at 37°C. Transduced cells were cultured for 2-3 days and analyzed by flow cytometry. For reconstitution experiments, single-cell suspensions were prepared from the freshly isolated bone marrow of RhoA*^fl/fl^* and RhoA*^fl/fl^* mb1 ^+/hcre^ mice. CD19^+^ cells were isolated from this single-cell suspension using the CD19 Microbeads based on the positive selection method (Miltenyi biotech) in an AutoMACS Pro separator. Sorted cells were cultured in Iscove’s medium containing 10% heat-inactivated FCS and IL7. After 3 days, cells were retrovirally transduced with an expression vector for *RhoA, Foxo1*, *Pten* or empty vector alone as the control. Stable growing cell lines of RhoA*^fl/fl^* bm-derived pre-B cells were retrovirally transduced with tamoxifen-inducible Cre-recombinase (ERT2-Cre) or empty control vectors and subsequently selected by addition of puromycin. Cre-recombinase was activated by the addition of 1- 2 μM 4-hydroxytamoxifen (4-OHT, Sigma Aldrich). As control cells were treated with EtOH (solvent of 4-OHT). *RhoA* deletion was analyzed 72 hours after 4-OHT inductions by FACS analysis, PCR or immunoblotting. The following primers were used to detect *RhoA* by PCR after deletion; m-RhoA-F TGC CAT CAG GAA GAA ACT CGT and m-RhoA-R CAA GAT GAG GCA CCC AGA CTT. The size of the respective band is 574 bp. Actin with a size of 400 bp was used as the loading control and detected using the following primers; m-Actin-F GATCACTATTGGCAACGAGC, m-Actin-R ACGCAGCTCAGTAACAGTCC

### Establishment of Murine BCR-ABL1+ Leukemia Cells

BM cells from *RhoA^fl/fl^ Mb1^fl/fl^* or *RhoA^fl/fl^ Mb1^Cre/Cre^* mice were cultured for 3-7 days in Iscove’s medium containing 10% heat-inactivated FCS, 2 mM L-glutamine, 100 U/ml penicillin/streptomycin, and 50µM 2-mercaptoethanol. The medium was also supplemented with the supernatant of J558L plasmacytoma cells stably transfected with a vector encoding murine IL7. The pro-/pre-B-cells were retrovirally transformed with a pMIG vector expressing BCR-ABL1. Transformed cells were selected by IL7 withdrawal (48).

### Ca^2+^ Measurement

Measurement of Ca^2+^ mobilization was previously described (45). Briefly, a total of 1 x10^6^ ER^T2^-SLP65, pre-BCR positive TKO cells were loaded with Indo-1 AM (Invitrogen) using Pluronic F27 (Invitrogen). ER^T2^-SLP65 function was induced by the addition of 2 µM 4-OHT or the respective amount of its solvent Ethanol (Et) as control. Pre-BCR expression and Ca2+ mobilization was acquired at a FACS LSR Fortessa flow cytometer (BD).

### Mice

All mouse housing, breeding, and surgical procedures were approved by the governmental institutions of Baden-Württemberg (Regierungspräsidium Tübingen).

#### Generation of RhoA^fl/fl^ Mice

Conditionally targeted Homozygous mice for the floxed *RhoA* allele (*RhoA^fl/fl^*) were generated as previously described (66). For a conditional *RhoA* deletion in early B cell lineage, *RhoA^fl/fl^* mice were crossed with mice expressing a variant of *hCre* recombinase under the control of the B cell specific *Mb1* gene promoter (67). For conditional deletion of *RhoA* at mature B cell stages, *RhoA^fl/fl^* mice were crossed with mice expressing Cre recombinase under the control of CD21 (50).

The mice were genotyped using JVH11 (5’-AGC CAG CCT CTT GAC CGA TTT A-3’) and JVH14 (5’-ATG TCA AAG AGG AAA TAC TGC-3’) primers resulting in 393bp band for *RhoA^fl/fl^* and 279bp band for *RhoA^+/+^*. Primers for Mb1^+/hCre^ hCre-For (5’-ACC TCT GAT GAA GTC AGG AAG AAC-3’) and hCre-Rev (5’-GGA GAT GTC CTT CAC TCT GAT TCT-3’) resulting in a 500bp for hCre. Primers for CD21^+/Cre^ CD21 NDE (5’-TCT GGC ATA CTT ATT CCC TGA AG-3’) and CD21 Cre rev (5’-GAA CCT CAT CAC TCG TTG CAT C-3’) resulting in a 450bp band for Cre.

#### Generation of Eµ-TCL1 RhoA^fl/fl^ Mb1^+/Cre-ERT2^ Mice

*Eμ-TCL1* mice (54) were crossed with Mb1^+/Cre-ERT2^ strain which expresses a tamoxifen-inducible form of the *Cre* recombinase under the control of the *Mb1* promoter region. Then, the mice were crossed with *RhoA^fl/fl^* mice to generate *Eµ-TCL1*, *RhoA^fl/fl^ Mb1^+/Cre-ERT2^* strain. All animals were maintained in accordance with the German Animal Welfare Act, which have been reviewed by the regional council and approved under the license (#1288 L1-L18). Mice were maintained for 10 months before starting the experiment. Mice were treated 3x every third day by oral gavage of 6mg tamoxifen (Ratiopharm) dissolved in 20% ClinOleic (Baxter) per mouse and sacrificed after 5 days from the last treatment. Control mice were left untreated.

The mice were genotyped using the primers Mb1cre-for (5’-ACA AAG GGG AAA GGG AAG AA-3’) and Mb1cre-rev (5’-CAT GTT TAG CTG GCC CAA AT-3’) resulting in a 500 bp band for the mb1-creER^T2^ allele. The primers hTCL1-for (5’-GGC CGA GTG CCC GAC ACT-3’) and hTCL1-rev (5’-CGA GAA GCA TGT CCT CCA CG-3’) resulted in a band of 300 bp indicating the ETCL-1 transgene. The primers JVH11 (5’-AGC CAG CCT CTT GAG GGA TTT A-3’) and JVH15 (5’-TGT GGG ATA GCG TTT GAG CAT-3’) were used to identify the RhoA-floxed allele at 393 bp and the RhoA-wt allele at 279 bp.

### *In Situ* Proximity Ligation Assay (PLA)

For PLA experiments (68) with SLP65, pSLP65, RHOA and GEF-H1 the corresponding antibodies were used (RHOA (26C4) santa cruz sc-418; BLNK (2B11) santa cruz sc-8003; Phospho-BLNK (Thr152) (E4P2P) Cell Signaling #62144; GEF-H1 (55B6) Cell Signaling #4076). The antibodies for RHOA and BLNK were labeled directly using the Duolink *in situ* Probemaker plus/minus reagent (Sigma-Aldrich). GEF-H1 and pSLP65 were detected by the corresponding secondary antibody Duolink *in situ* PLA Probe Anti-Rabbit plus/minus (Sigma-Aldrich).

The PLA probes were then subjected to ligation and polymerization reactions using the Duolink *in situ* detection reagents orange (Sigma-Aldrich). In the end, the cells were examined for the frequency of signals per cell under the fluorescence microscope (Leica). Pictures were taken and quantified by Image J software.

### G-LISA Assay

TKO cells reconstituted with an expression vector for the pre-BCR and inducible form of SLP65 was used for the study. Cells were serum starved for 48 hours and were either stimulated with 1µM 4-OHT or its control EtOH for different time points. After stimulation cells were then snap-frozen in liquid nitrogen and GTP bound RHOA level was measured using GLISA-Kit (Cytoskeleton, Inc) according to manufacture’s protocol.

### RHOA Inhibition

Bone marrow cells derived from wild type mice were cultured in Iscove’s medium supplemented with IL7 for 5 days. Cells were serum starved for 3 hours before treating with different concentrations of RHO Inhibitor (CT04, Cytoskeleton, Inc). After 16 hours cells were collected, washed and lysed with RIPA buffer containing Protease and Phosphatase inhibitor cocktail. The amounts of protein in the lysates were determined using Bradford assay and proteins of interest were detected using immunoblotting.

### Immunoprecipitation

DG75EB/HA-RhoA cells were serum starved for 30 mins and stimulated for 0,1,3,5 and 10 mins with 10 µg/ml anti-human IgM F(ab)’2 (Southern biotech) at 37°C in a thermomixer. Reactions were stopped by adding ice-cold PBS and cells were lysed using co-immunoprecipitation lysis buffer (I mM EDTA, 50 mM Tris pH-7.5, 150 mM NaCl, 0.1% Triton X-100 together with Protease and Phosphatase inhibitor cocktail). Immunoprecipitation of cell lysates was performed using Protein G Sepharose 4 Fast Flow 50% gel suspension (GE health care) with an anti-BLNK antibody according to the manufacture’s instructions. Precipitated proteins were detected using immunoblotting.

### Immunoblotting

Equal amounts of denatured protein were separated on a 10% gel by SDS-electrophoresis and transferred onto a PVDF membrane. The membrane was incubated for 1 hour with blocking buffer (TBS/0.1% Tween-20; Sigma) with 5% BSA (Serva) and incubated overnight with the primary antibody diluted in blocking solution. Antibodies used for immunoblotting are listed in **Table 2**.

**Table 2:** Antibodies used for Immunoblotting.

Antigen	Origin	Clone	Company
GEF-H1	Rabbit	55B6	Cell signaling Technology
pGEF-H1 (S886)	Rabbit	E1L6D	Cell signaling Technology
BLNK (2B11)	Mouse	SC-8003	Santa Cruz Biotechnology
BLNK	Rabbit	DP32H	Cell signaling Technology
p-BLNK	Rabbit	E4P2P	Cell signaling Technology
Plcg2	Mouse	H-160	Santa Cruz Biotechnology
p-plcg2	Rabbit	Y759	Cell signaling Technology
GAPDH	Rabbit	14C10	Cell signaling Technology
RhoA	Mouse	SC-418	Santa Cruz Biotechnology
RhoA	Rabbit	67B9	Cell signaling Technology
FOXO1	Rabbit	C29H4	Cell signaling Technology
p-FOXO1(s256)	Rabbit	S256	Cell signaling Technology
AKT	Rabbit	67E7	Cell signaling Technology
p-AKT (s473)	Rabbit	D9E	Cell signaling Technology
PTEN	Rabbit	D4.3	Cell signaling Technology
Anti-Mouse IgG	Horse		Cell signaling Technology
Anti-Rabbit IgG	Goat		Cell signaling Technology

After washing steps, the membrane was incubated for 1 hour at room temperature (RT) with horseradish peroxidase (HRP)-coupled secondary antibodies diluted in blocking buffer. Membranes were washed off to remove excess antibody and stained proteins were detected with an ECL Ultra Solution kit (Lumigen) on a Fusion SL advanced imaging system (Vilber Lourmat).

### Microscopy

SLP65-deficient pre-B cells were retrovirally reconstituted with ER^T2^-SLP65 and were then induced with 4-OHT for 30 min with or without Rho-specific inhibitor C3-toxin (RHOA I). Cells treated with vehicle were used as a control. Cells were then fixed with 2% paraformaldehyde and labeled with DyLight 488 conjugated anti-PTEN antibody and DAPI staining for immunofluorescent microscopy. Images were captured using a Zeiss LSM 780 with GaAsP detectors laser scanning confocal microscope with a 63× 1.4-NA Plan-Apochromat oil immersion objective. Images were taken at z-sections (15–20 sections) of 0.5μm intervals by using the 488nm Argon laser and 405nm diode laser for Alexa 488 and DAPI visualization, respectively. To avoid bleed-through effects in double triple-staining experiments, each dye was scanned independently in a multitracking mode, with the emission of DAPI being collected between 410-490nm and of Alexa 488 between 500-550nm. Clustered PTEN and 3D surface rendering, was determined using Imaris software (Bitplane, Inc.).

### Flow Cytometry

For flow cytometric analysis, cell suspensions were pretreated with anti-CD16/CD32 Fc-Block (2,4G2; BD Biosciences) and stained by standard procedures. Dead cells were excluded by staining with Fixable Viability Dye eFluor780 (eBioscience). Cells were stained by using antibodies enlisted in **Table 3**.

**Table 3:** Antibodies used for flow cytometry.

Antigen	Specificity	Conjugate	Clone	Company
B220/CD45R	Mouse/Human	Pe-cy7/PerCP-eFluor 710	RA3-6B2	eBioscience
Blimp-1	Mouse	Alexa Fluor 647	5E7	BioLegend
CD3e	Mouse	Pe-Cy7	145-2c11	eBioscience
CD5	Mouse	PE	53-7.3	eBioscience
CD11b	Mouse	PE	M1/70	eBioscience
CD19	Mouse	PerCP-Cy5.5/eFluor 450/APC	ID3	BD Biosciences/eBioscience
CD21/CD35	Mouse	APC	7E9	eBioscience
CD23	Mouse	PE	B3B4	eBioscience
CD25	Mouse	APC	PC61	BD Biosciences
CD43	Mouse	PE or FITC	S7	BD Biosciences
CD117	Mouse	PE	ACK45	BD Biosciences
CD138	Mouse	PE	DL-101	eBioscience
CD138	Mouse	Brilliant Violet 421	281-2	BioLegend
Kappa	Mouse	PE	Polyclonal	Southern Biotech
IgD	Mouse	APC	11-26	eBioscience
IgD	Mouse	FITC	11-26	Southern Biotech
IgM	Mouse	eF-450	eB121-15-F9	eBioscience
IgM	Mouse	FITC	Polyclonal	Southern Biotech
IgM	Mouse	Pe-Cy7	2/41	eBioscience
CD16/CD32	Mouse		2.4 G2	BD Biosciences

Intracellular staining for flow cytometry was performed after surface staining by using the True-Nuclear Transcription Buffer Staining Set (BioLegend) according to the manufacturer’s instructions.

Cells were acquired at a FACSCantoII flow cytometer (BD Biosciences). If not indicated otherwise, numbers in the dot plots indicate the percentages of cells in the respective gates, numbers in the histograms indicate the mean fluorescence intensity (MFI).

### Expression Assays

Total RNA was isolated using ReliaPrep™ RNA Cell Miniprep System (Promega), and PicopureTM RNA isolation kit (Arcturus). cDNA was synthesized using High capacity RNA to cDNA kit (Applied Biosystems) and RevertAid Reverse Transcriptase kit (Thermo Fischer). Quantitative real-time PCR (qRT-PCR) analyses were performed using TaqMan PCR probe (GAPDH: Mm99999915_g1, RAG1: Mm01270936_m1, RAG2: Mm01270938_m1, Applied Biosystems) with TaqMan gene expression mastermix (Applied Biosystems). qRT-PCR data were acquired on a StepOnePlus Real-Time thermocycler (Applied Biosystems) and analyzed with the StepOne Software version 2.3. Relative quantification (RQ) was calculated using the 2^-ΔCCT equation.

### Immunohistochemistry

For cryosections, spleens were embedded in O.C.T.-compound (SAKURA) and frozen at -80°C. Five μm sections were prepared using a cryo-microtome (Reichert-Jung 2800 Frigocut) with a S35 knife (Feather) and fixed on SuperfrostPLUS slides (Thermo Scientific) by treatment with pure acetone. Prior to staining, the sections were rehydrated with PBS + 2% BSA + 0.1% sodium azide and blocked with Fc-Block (anti-CD16/32; BD Biosciences). The section was mounted with Fluoromount-G (Southern Biotech) after staining with FITC conjugated anti-CD169 (MOMA1, AbD Serotec) and Cy5 conjugated anti-IgM (Jackson Immunoresearch) antibodies. The staining was detected using a DMi8 fluorescence microscope (Leica).

### Histology of Kidney

Kidneys from *RhoA^fl/fl^* or *RhoA^+/fl^* (N=3) and *RhoA^fl/fl^ Cd21^+/Cre^* (N=6) mice of age 33-49 were embedded in O.C.T.-compound (SAKURA), shock frozen in liquid nitrogen and stored at -80°C. Two µm sections were prepared using a cryo-microtome (Leica cryostat CM1950) with a C35 microtome blade (Feather). Sections were mounted on SuperfrostPLUS slides (Thermo Scientific) and fixed within pure acetone (Sigma Aldrich) for 9 min. Sections were stained with HE stain (Waldeck GmbH & Co.) and PAS stain (Sigma Aldrich). The slides were blinded and the sections were examined independently by two senior pathologists for pathological changes in the general cellularity and architectural features of the kidney.

### Proteinuria

Urine samples were collected from mice (age 23-48 weeks) of the indicated genotype and proteinuria levels were measured using Combur-Test ^®^ strips (Roche).

### Generation of BCP-ALL Xenografts

One million cells/animal were injected intravenously into black6 *γc ^-/-^ Rag2 ^-/-^* mice and leukemia engraftment was followed by detection of GFP^+^ cells in the peripheral blood *via* flow cytometry analysis. Animals were sacrificed after 14 days upon showing clinical signs of leukemia including loss of activity, organomegaly, and hindlimb paralysis. Leukemic infiltration of the murine CNS was assessed using histological sections in blinded experiments (49).

### Immunization

*RhoA^fl/fl^* (N=7) and *RhoA^fl/fl^ Cd21^+/Cre^* (N=8) mice of 6-8 weeks old were immunized with 100 µg NP(24)KLH (Biosearch Technologies California, N-5060-5) + 100µg CpG-ODN1826 (Biomer, custom design) in PBS or with 100µg CpG-ODN1826 (control immunization). Sera were collected at days 7, 14 and 21 post-primary immunization (day 0) and after 7 and 35 days after booster immunization (day 21). Then IgM and IgG antibodies to NP were measured by NP-BSA (Biosearch Technologies California, N-5050L-10) ELISA fitted concentration in arbitrary units (AU) according to a standard (SouthernBoitech). Mice from which no blood were withdrawn were excluded from the analysis. The decline of anti-NP-IgG titers was calculated as the ratio of IgG titers at day 28 relative to day 56.

### Enzyme-Linked Immunoblot Assay (ELISA)

100 µl of mouse blood samples were collected from living mice by a transverse incision through the major tail vein, and incubated in an Eppendorf tube containing heparin, the samples were centrifuged for 15min at 1400 rpm at 4°C. For the anti-IgM and anti-IgG ELISAs, 96-well plates (NUNC, maxisorp) were coated with polyclonal anti-mouse IgM or IgG antibody (SouthernBiotech) and blocked with buffer containing 1% BSA. Dilutions of anti-mouse IgM/IgG (SouthernBiotech) were used as standard. The concentration of IgM and IgG in the supernatants was determined by detection with alkaline-phosphatase-labeled anti-mouse IgM or IgG (Southern Biotech), respectively. P-nitrophenylphosphate (Genaxxon) in diethylamine buffer was added and data were acquired at 405 nm using a Multiskan FC ELISA plate reader (Thermo Scientific). To determine the content of autoreactive anti-dsDNA specific antibodies, concentration-adjusted cell culture supernatants were applied to plates coated with calf thymus DNA (Rockland Immunochemicals).

### Migration Assay

5 × 10^5^ cells were cultured on the top chamber of a transwell culture insert (Corning, pore size 5 μm) and were allowed to migrate toward media containing 100 ng/ml CXCL12 (ImmunoTools) for 16 h. The total cell number in the lower chamber was determined using hematocytometer.

### *In Vitro* Proliferation Assay

The proliferation dye (eFluor 670; eBioscience) was used to label cells which were cultured in optimum conditions as described previously (69). The percentage of proliferating cells were determined by flow cytometry after 72 hours.

### Statistics and Reproducibility

Statistical tests are indicated in the figure legends. Results were analyzed for statistical significance with GraphPad Prism 8.3.0 software or SPSS (v 24.0.0.2). A *p* value of <*0.0500* was considered significant (*p<0.005, **p < 0.001, ***p < 0.001, ****p < 0.001). *In vitro* panels are representative of at least 3 independent experiments, unless mentioned otherwise.

## Data Availability Statement

The original contributions presented in the study are included in the article/**Supplementary Material**. Further inquiries can be directed to the corresponding author.

## Ethics Statement

The animal study was reviewed and approved by Regierungspräsidium Tübingen.

## Author Contributions

AV, HF, IO, HA, CS, AK, and MB analyzed *RhoA*-deficient mice. TA performed immunization experiments. AV and HA performed *in vitro* deletion and re-constitution experiments. MB and AK measured Igs in serum and urine protein. JB analyzed histology sections of kidney from mice. AS analyzed CNS histology sections of murine ALL mice. NE and CH generated DG75-EB *RhoA*-KO cells. AK and OE measured RHOA activity *in vitro*. OE performed protein expression analysis in different B cell types. VS and EH performed PLA and generated and analyzed Eµ-TCL1 RhoA^fl/fl^ *Mb1^+/Cre-ERT2^* mouse. CB generated *RhoA* ^fl/fl^ mouse. AAlk performed experiments. AV contributed in writing the manuscript. MD analyzed the data, prepared figures and wrote and revised the manuscript. AAls provided BCR-ABL1^+^ ALL *in vivo* data, prepared figures and wrote the manuscript. EH and AAls designed experiments and discussed the research direction. HJ initiated, designed, supervised research and wrote the manuscript. All authors discussed the manuscript. All authors contributed to the article and approved the submitted version.

## Funding

This work was supported by the DFG through TRR130 (B cells and beyond) project 01, SFB1074 (Experimental Models and Clinical Translation in Leukemia), SFB 1279 (Exploration of the Human Peptidome), JU 463/5-1, and ERC advanced grant (694992).

## Conflict of Interest

The authors declare that the research was conducted in the absence of any commercial or financial relationships that could be construed as a potential conflict of interest.

## Publisher’s Note

All claims expressed in this article are solely those of the authors and do not necessarily represent those of their affiliated organizations, or those of the publisher, the editors and the reviewers. Any product that may be evaluated in this article, or claim that may be made by its manufacturer, is not guaranteed or endorsed by the publisher.
