# Supplementary material for: The Small GTPase RHOA Links SLP65 Activation to PTEN Function in Pre B Cells and Is Essential for the Generation and Survival of Normal and Malignant B Cells
Source: Front Immunol. 2022 Mar 15;13:842340. doi: 10.3389/fimmu.2022.842340 (PMC8965026; doi:10.3389/fimmu.2022.842340)

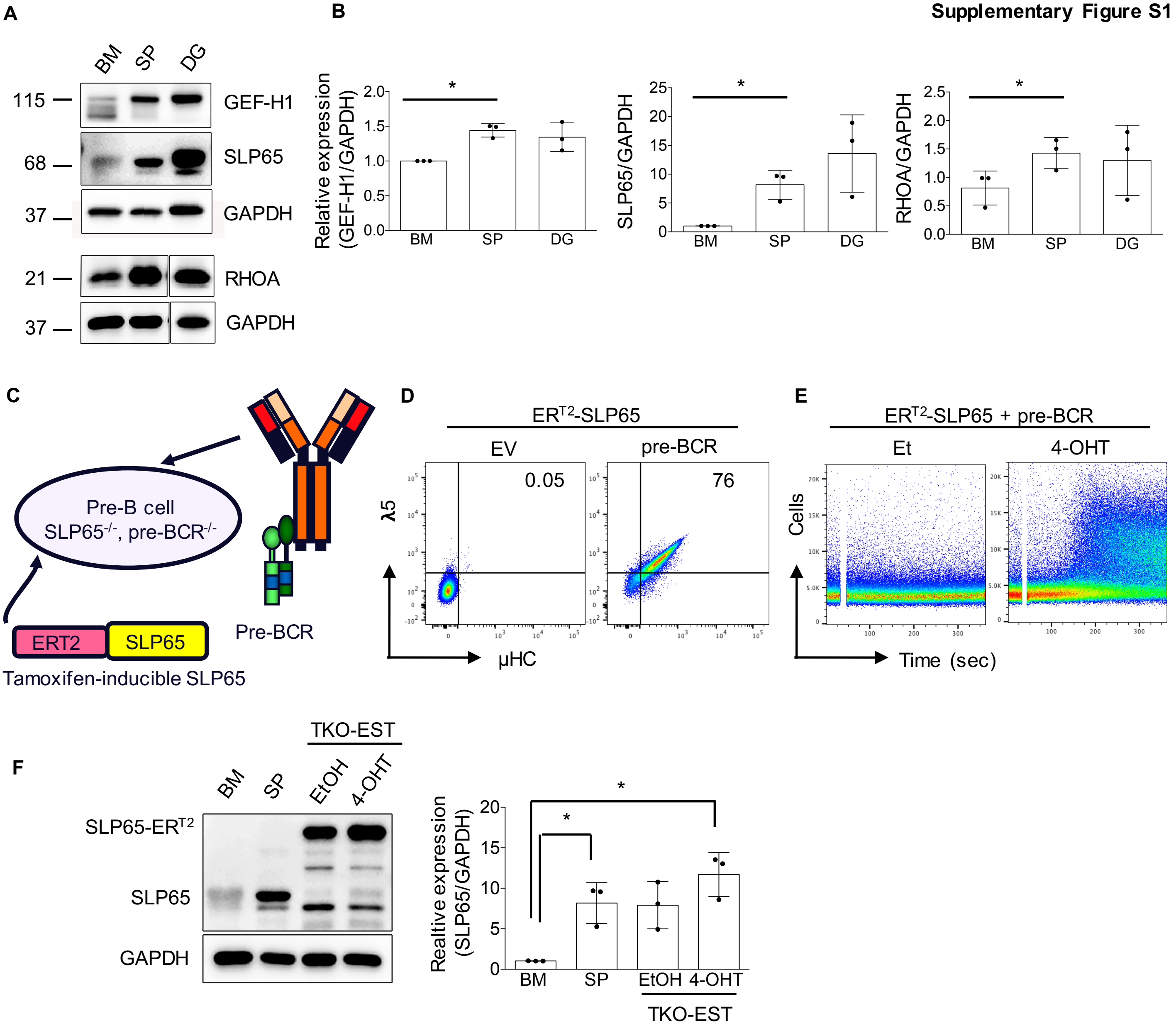

Supplementary Figure 1: Expression of SLP65, GEF-H1 and RHOA in different B cells. A. Western blot showing expression of GEF-H1, SLP65 and RHOA in mouse primary B cells derived from bone marrow (BM), spleen (SP) and DG75EB/HA-RhoA cells. GAPDH is used as loading control. B. Quantification of the western blots normalized with respect to GAPDH. Bars represent mean ± SD. Statistical test- one-way ANOVA with multiple comparison test, n=3. C. Schematic representation of the inducible SLP65 expression system. Bone marrow derived early pre-B cells (*Rag2*$^{-/-}$, *λ5*$^{-/-}$, *Slp65*$^{-/-}$ also called TKO) were reconstituted with μHC, λ5 and inducible SLP65 (TKO-EST). (D-E). Surface expression of pre-BCR components after reconstitution (D) and Calcium mobilization after activation of ER^{T2}-SLP65 fusion protein by 4-hydroxytamoxifen (4-OHT; 2μM) (E). The data represent at least three independent experiments. F. Left panel- western blot showing expression of SLP65 in mouse primary B cells (BM and SP) and TKO-EST cells upon SLP65 activation. GAPDH is used as loading control. Right panel- quantification of the western blots normalized with respect to GAPDH. Bars represent mean ± SD. Statistical test- one-way ANOVA with multiple comparison test, n=3.

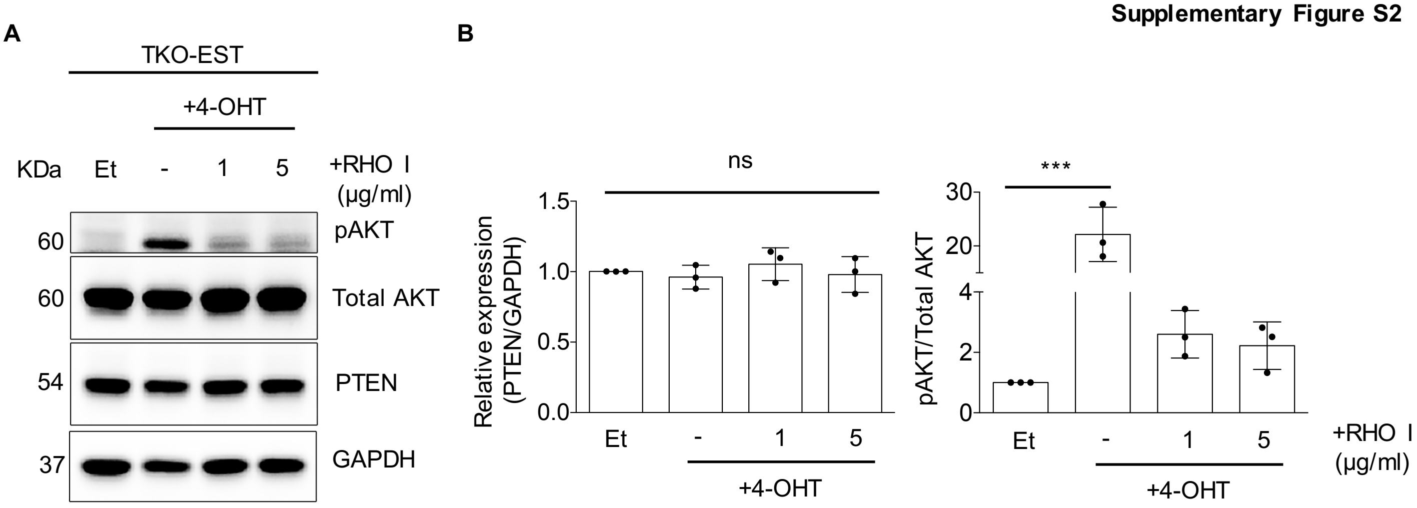

Supplementary Figure 2: AKT activation and PTEN expression in TKO-EST cells upon RHO inhibition . A. Western blot showing expression of phosphorylated AKT, total AKT and PTEN in TKO-EST cells upon RHO inhibition. The cells were treated with RHO inhibitor for 16 hours followed by treatment with 4-OHT to activate SLP65. Cells treated with ethanol (Et) is used as control. GAPDH is used as loading control. B. Quantification of the western blots. PTEN is normalized with respect to GAPDH and phospho AKT is normalized with respect to total AKT. Bars represent mean ± SD. Statistical test- one-way ANOVA with multiple comparison test, n=3.

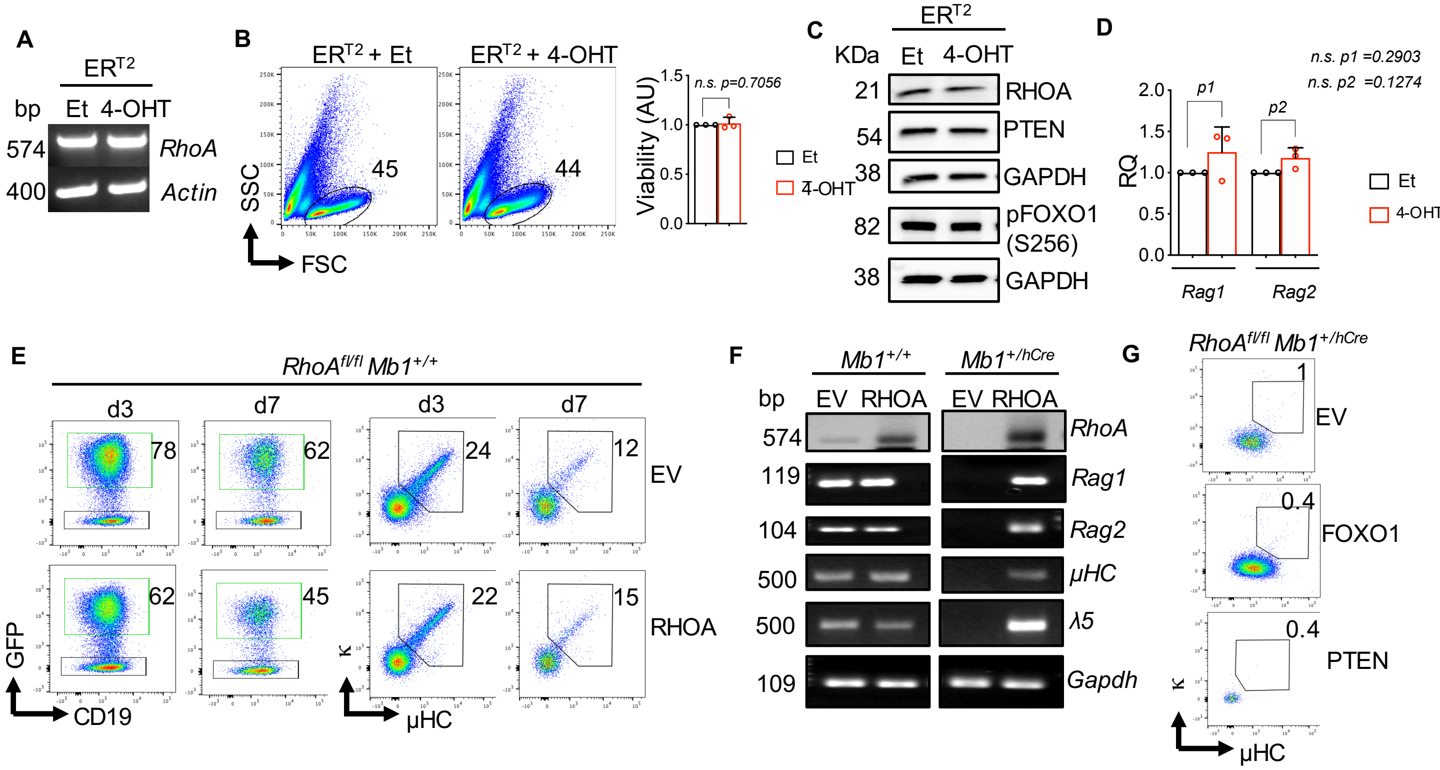

Supplementary Figure 3: Generation of *RhoA$^{fl/fl}$* cells transduced with ER^{T2}. *RhoA$^{fl/fl}$ Mb1$^{+/+}$* pre-B cells were transduced with ER^{T2}. Cells were then treated with either Tam or Et. A. RT-PCR showing *RhoA* gene expression after 2 days of tam induction. *Actin* was used as a loading control. bp: base pair. B. Left- the percentage of living cells were determined by flow cytometry after 3 days of tamoxifen induction. Right- the fold change of living cells after treatment with either ethanol or tamoxifen at day 3. N=3 independent samples per group, and error bars represent mean ± SD. Paired t-test, two-tailed. C. Western blot showing expression of RHOA, PTEN and phosphorylated FOXO1 after 2 days of tamoxifen induction. D. Quantitative RT-PCR showing expression of *Rag1* and *Rag2* after tamoxifen induction for 2 days. N=3 independent samples per group, and error bars represent mean ± SD. Paired t-test, two-tailed. E. *RhoA$^{fl/fl}$ Mb1$^{+/+}$* pre-B cells were reconstituted with either pMIG EV or pMIG-RHOA. Left- Flow cytometric analysis showing enrichment of *RhoA$^{fl/fl}$ Mb1$^{+/+}$* cells transduced with either an EV or RHOA (GFP^{+} population) after 3 and 7 days of transduction. Right- Flow cytometric analysis showing the percentage of differentiated cells (μ^{+}kappa^{+}) in *RhoA$^{fl/fl}$ Mb1$^{+/+}$* pre-B cells which were reconstituted with EV or RHOA (N=4). F. RT- PCR analysis for *RhoA, Rag1, Rag2, μ* and *λ5* genes in *RhoA$^{fl/fl}$ Mb1$^{+/+}$* and *RhoA$^{fl/fl}$ Mb1$^{+/hCre}$* pre-B cells 5 days after reconstitution with EV or RHOA. *Gapdh* was used as a loading control. G. Flow cytometric analysis showing the percentage of differentiated cells (μ^{+}kappa^{+}) in *RhoA$^{fl/fl}$ Mb1$^{+/hCre}$* pre-B cells which were reconstituted with EV, FOXO1 or PTEN after 7 days of transduction (n=2).

Supplementary Figure S4

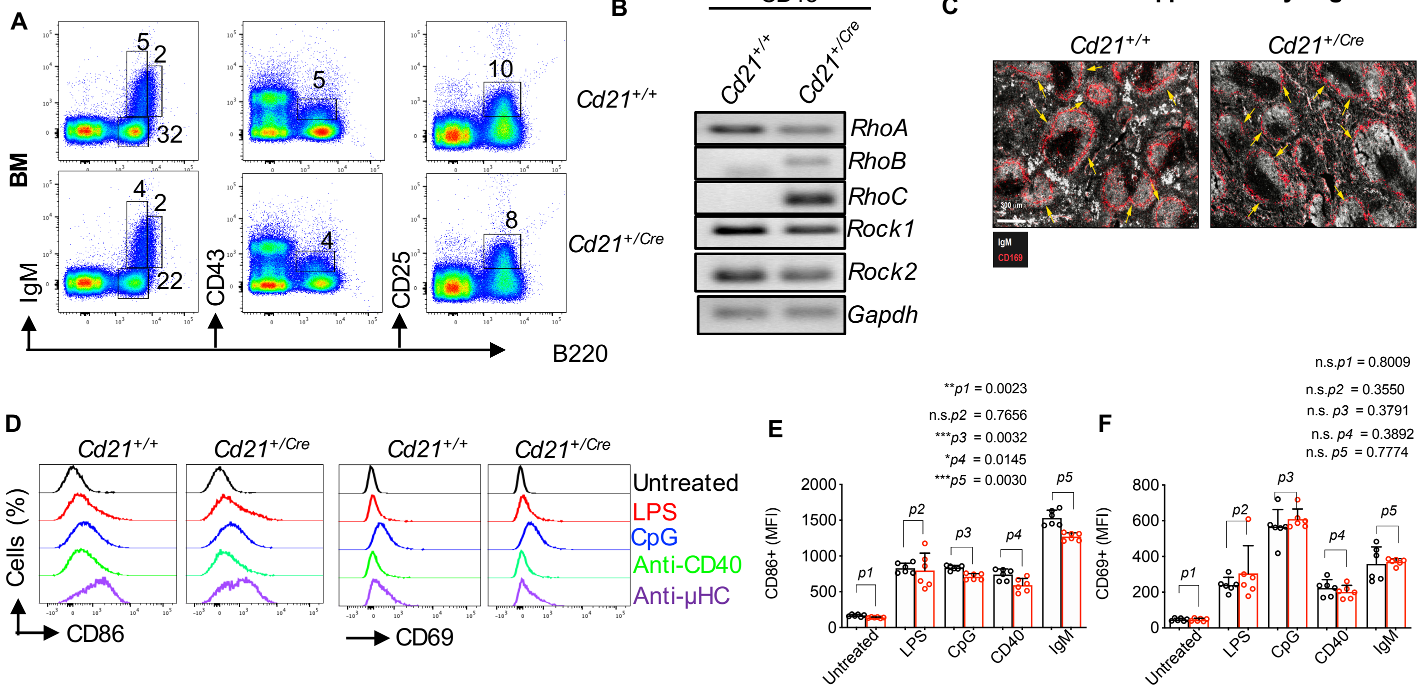

Supplementary Figure 4: Normal B cell development in the bone marrow of *RhoA*$^{fl/fl}$ *Cd21*$^{+/Cre}$ mice

A. Freshly isolated cells from bone marrow of *RhoA*$^{fl/fl}$ *and RhoA*$^{fl/fl}$ *Cd21*$^{+/Cre}$ mice were analyzed by flow cytometry for surface expression of the indicated markers. Data are representative of 3 mice. B. Splenic B cells from the corresponding mice were negatively MACS sorted using CD43 beads and expression of *RhoA, RhoB, RhoC, Rock1* and *Rock2* were measured by RT-PCR. *Gapdh* is used as loading control. C. Representative immunohistochemistry image of spleen sections from *RhoA*$^{fl/fl}$ and *RhoA*$^{fl/fl}$ *Cd21*$^{+/Cre}$ mice stained for CD169 (red) and IgM (white) at 10x magnification. Shown pictures are representative of 2 mice per genotype. Yellow arrows indicate marginal zone B cells. D. Flow cytometric analysis of CD86 and CD69 expression in splenic B cells derived from *RhoA*$^{fl/fl}$ and *RhoA*$^{fl/fl}$ *Cd21*$^{+/Cre}$ mice and treated *in vitro* with 10μg/ml LPS, 2.5μM CpG, 10μg/ml anti-CD40, or 10μg/ml anti-μHC for 24 hours. Cells were pre-gated on CD19^{+} cells. E-F. Quantification of mean fluorescence intensity (MFI) of CD86 (E) and CD69 (F) in the respective samples for the indicated treatments. N=6, and error bars represent mean ± SD. Unpaired t-test, two-tailed.

Supplementary Figure S5

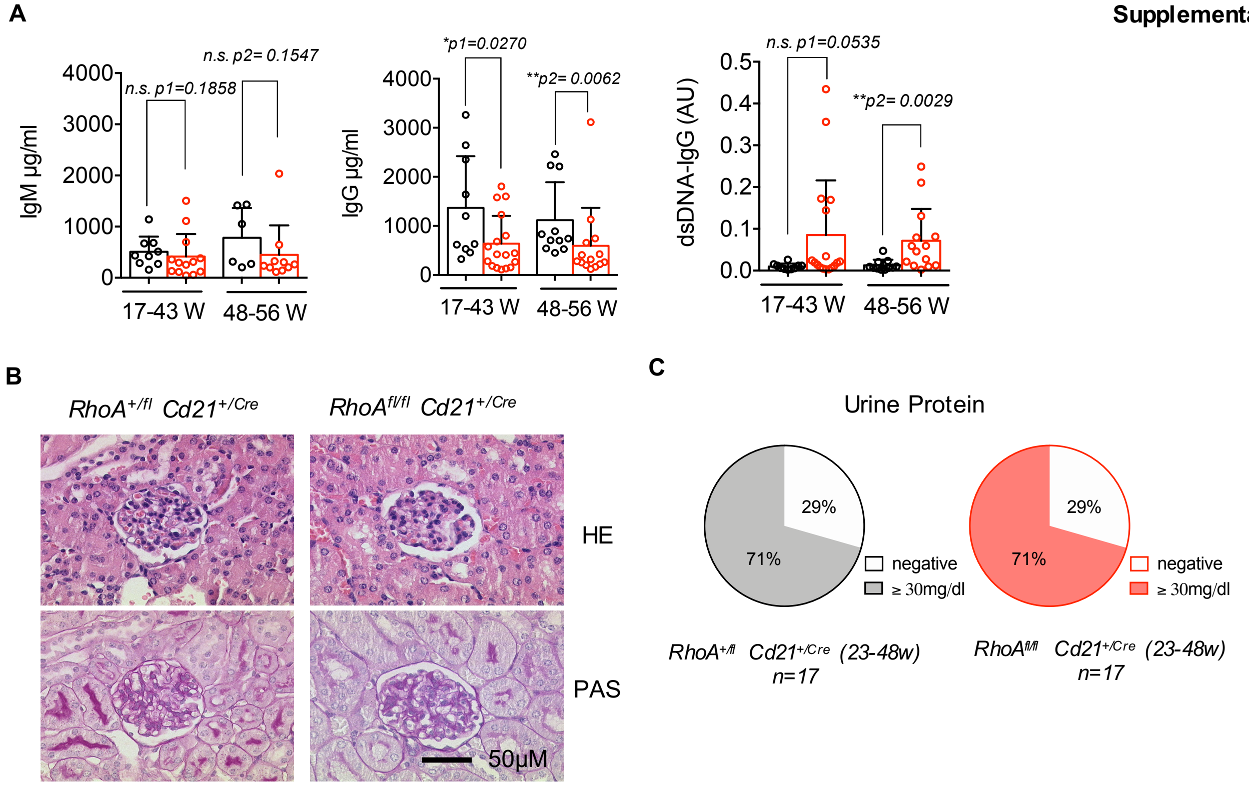

Supplementary Figure 5: Absence of autoimmunity in aged *RhoA*-deficient B cells. A. Serum levels of total IgM and IgG and autoantibodies against double stranded DNA in *RhoA*$^{fl/fl}$ (N=11-12) and *RhoA*$^{fl/fl}$ *Cd21*$^{+/Cre}$ (N=22-24) mice at the indicated age as detected by ELISA. B. Representative sections of renal cortex of *RhoA*$^{fl/fl}$ or *RhoA*$^{+/fl}$ (N=3) and *RhoA*$^{fl/fl}$ *Cd21*$^{+/Cre}$ (N=6) mice. Centrally depicted renal corpuscles and the surrounding renal tubules showed no pathological abnormalities (top: HE-staining, bottom: PAS-staining, 400x magnification). C. Proteinuria was measured in *RhoA*$^{fl/fl}$ (N=17) and *RhoA*$^{fl/fl}$ *Cd21*$^{+/Cre}$ (N=17) mice of the indicated age group and the percentage of high proteinuria was calculated. Proteinuria less than 30mg/dl was considered negative.

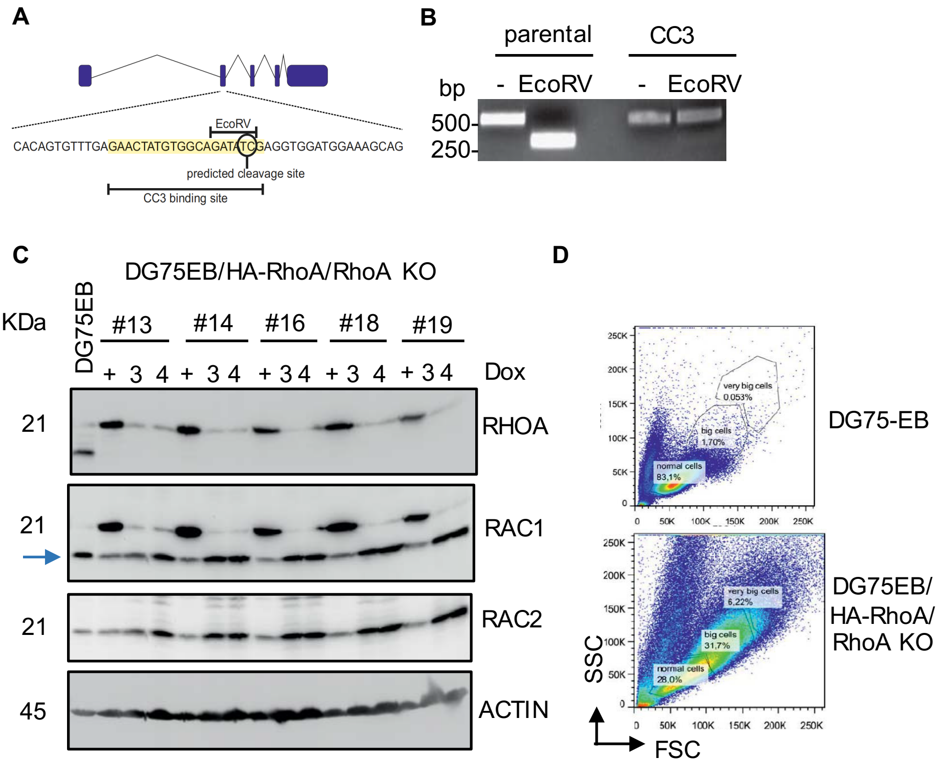

Supplementary Figure 6: RHOA is required for malignant B cells. A. The target sequence for CRISPR/Cas9 plasmid against *RHOA* which was introduced into DG75EB/HA-*RhoA* cells. The graph shows exon structure of the *RHOA* gene and sequence of the targeted exon 2. Binding site of the guide RNA is highlighted in yellow. B. CRISPR/Cas9 activity test. Exon 2 was amplified from parental DG75EB cells or CRISPR-targeted cells (CC3) and treated with EcoRV. Treated PCR product was tested by agarose gel electrophoresis. C. Western blot showing the deletion of RhoA upon removal of doxycycline (Dox) from DG75EB/HA-*RhoA* cells transduced with inducible *RhoA* CRISPR/Cas9 constructs. Doxycycline was removed for 3 or 4 days. Expression level is compared with the +Dox for each subclone and also the parental DG75EB cells. (d) Flow cytometric analysis of RhoA deleted cells showing their increased cell size after 4 days of doxycycline withdrawal.

Original Western blots

Figure 1A

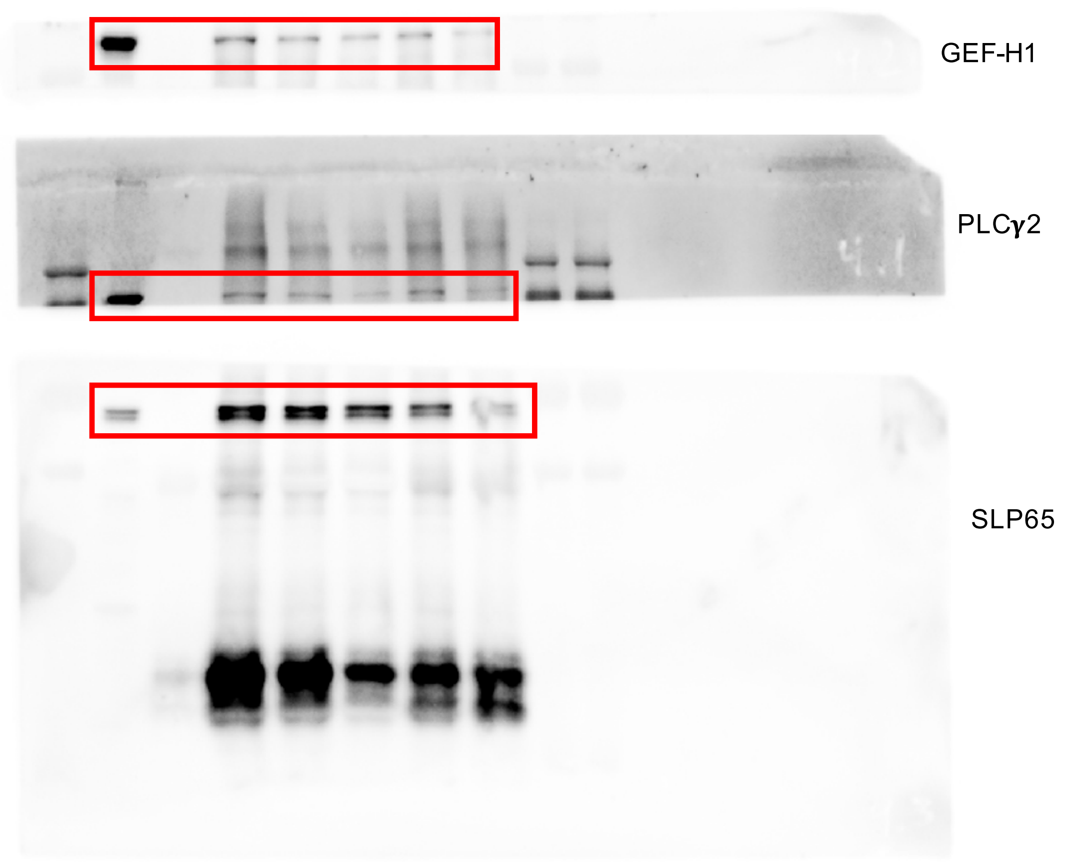

Figure 1D

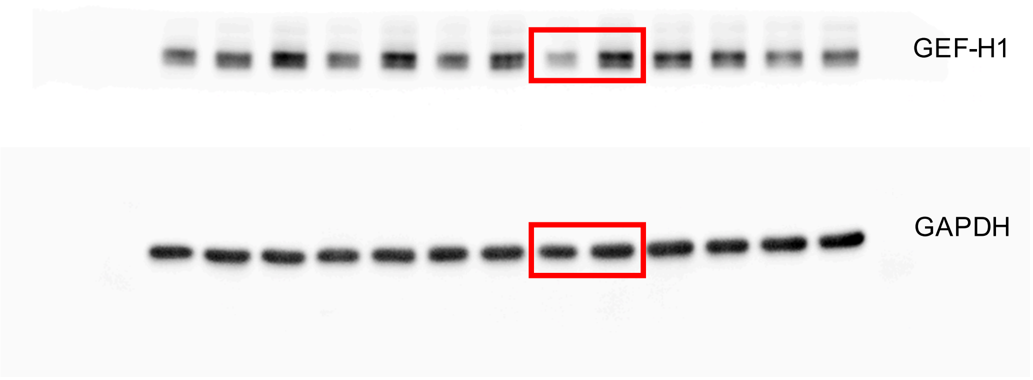

Figure 2D

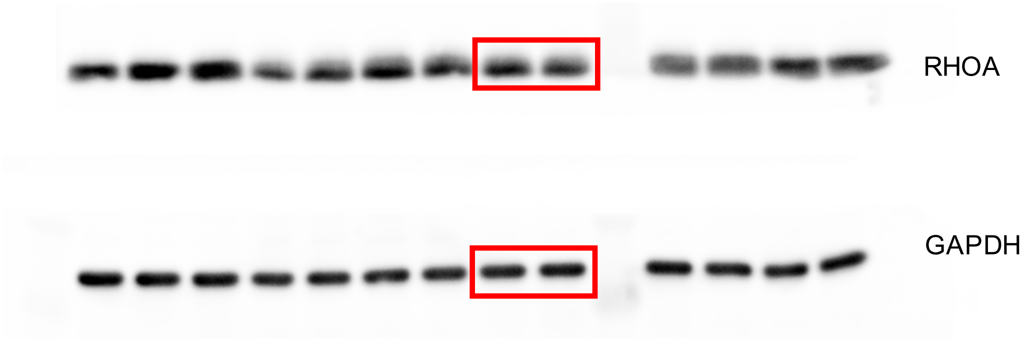

Original Western blots

Figure 3A

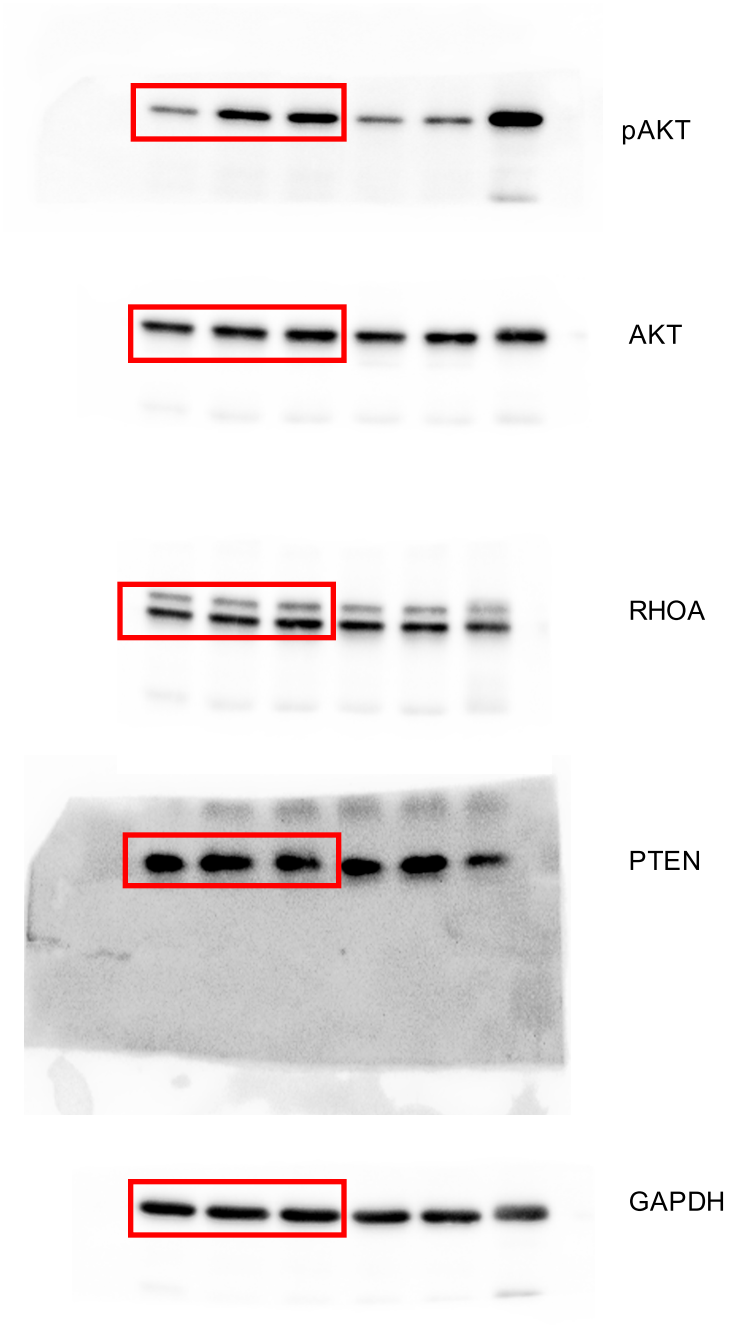

Figure 5C and Supplementary Figure 3C

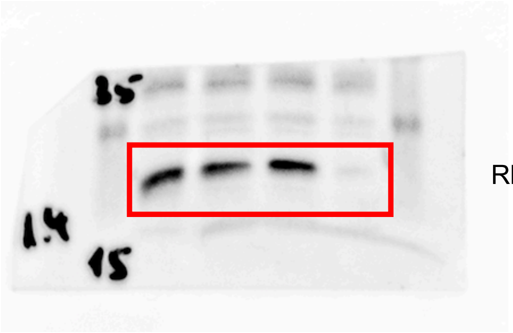

RHOA

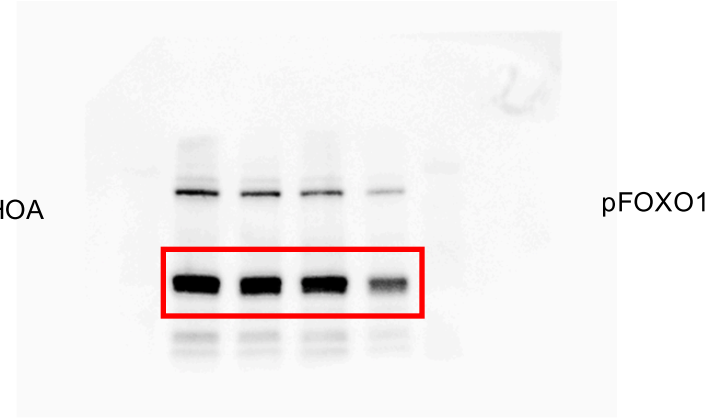

pFOXO1

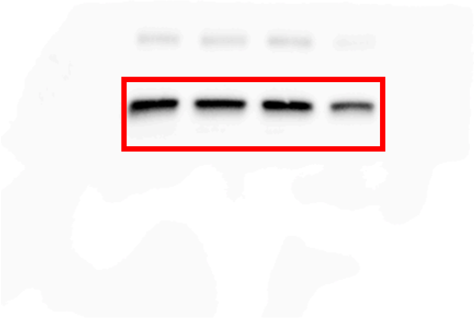

PTEN

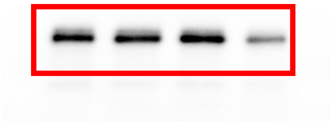

FOXO1

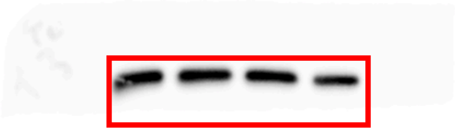

GAPDH

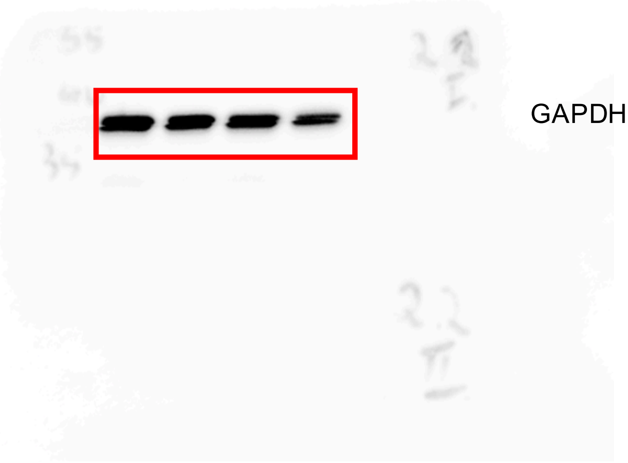

GAPDH

Original Western blots

Supplementary Figure 1A

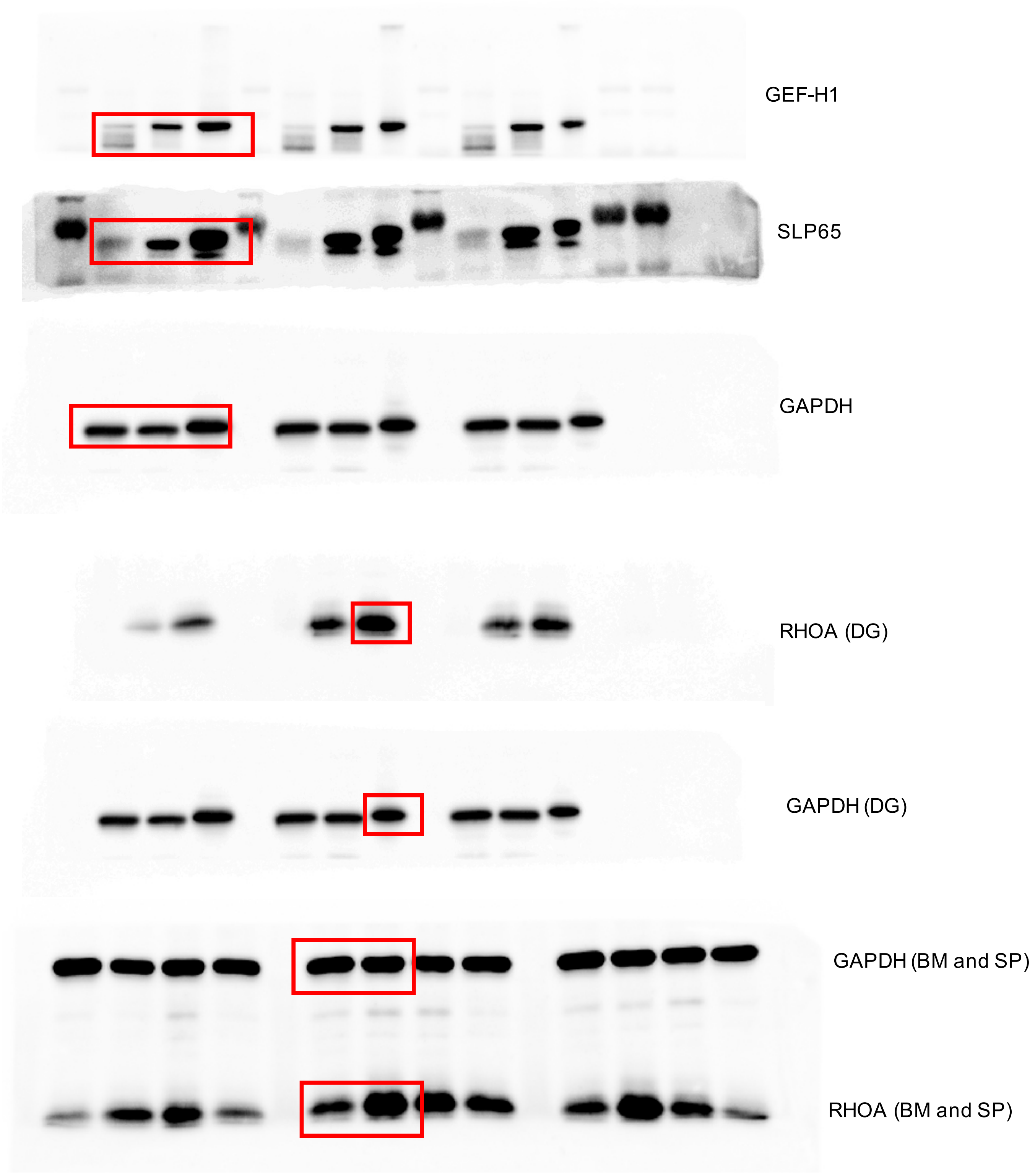

Supplementary Figure 1F

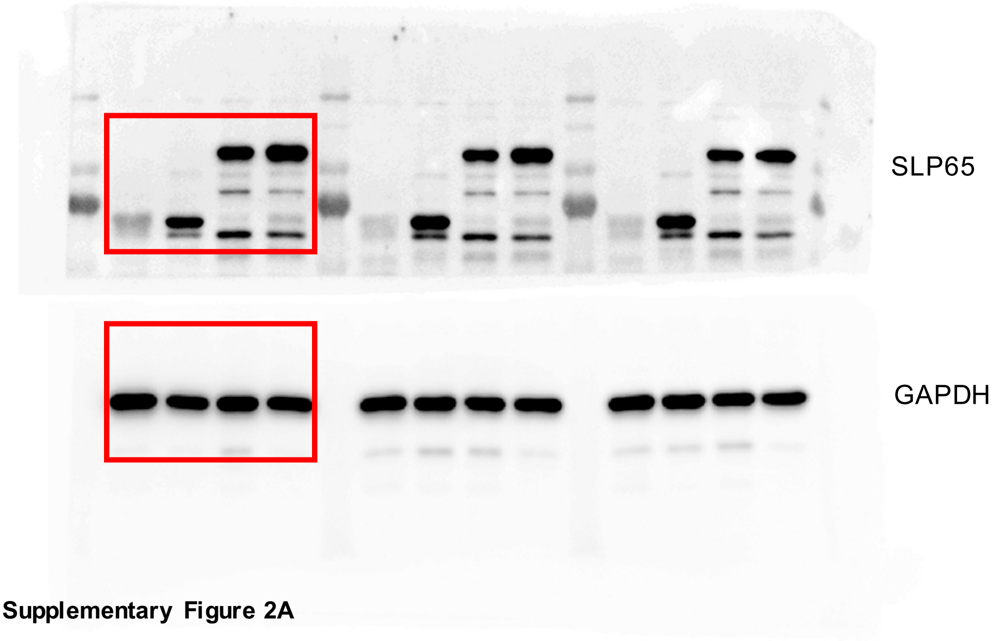

Supplementary Figure 2A

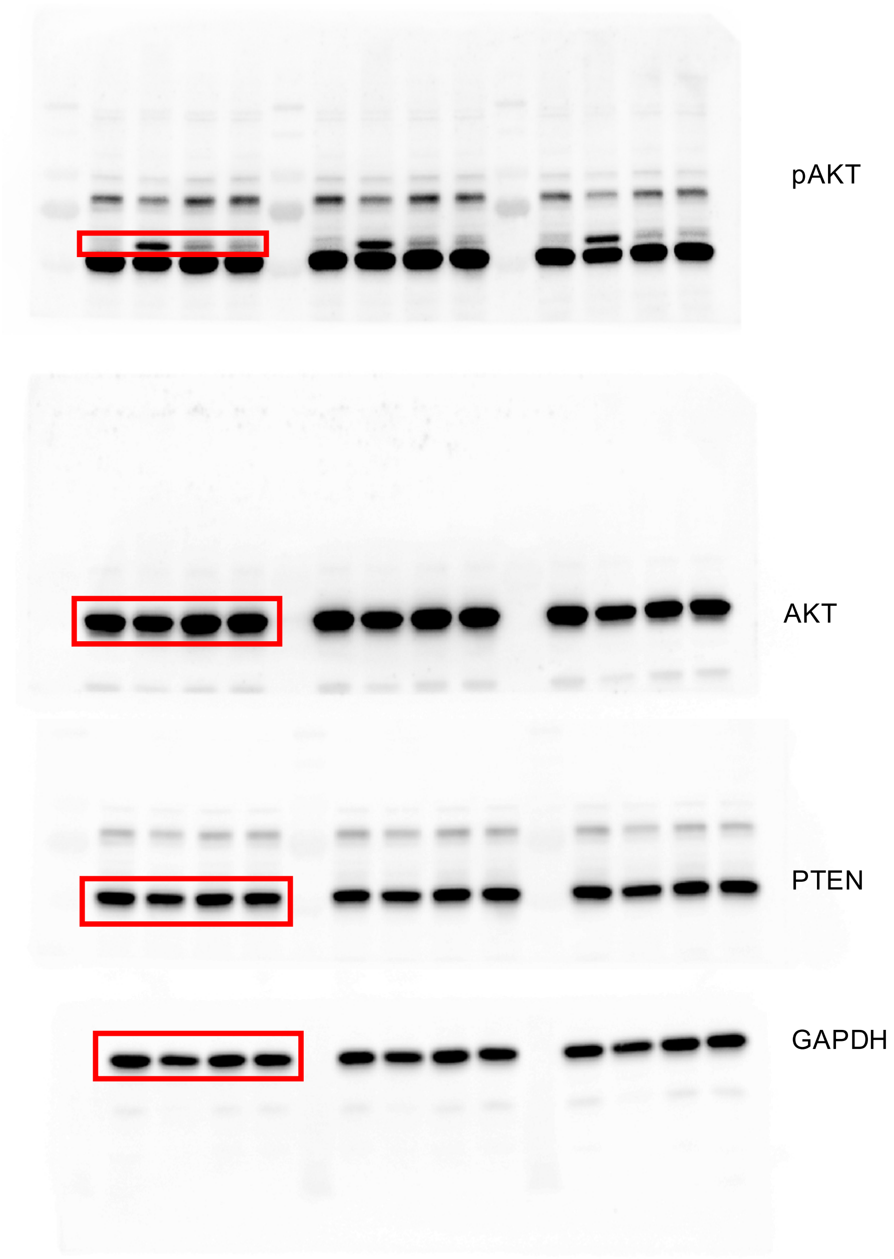

Supplement: Supplementary file 1 [file DataSheet_1.pdf]
